# K_2_S_2_O_8_-Promoted Aryl Thioamides Synthesis from Aryl Aldehydes Using Thiourea as the Sulfur Source

**DOI:** 10.3390/molecules23092225

**Published:** 2018-09-01

**Authors:** Yongjun Bian, Xingyu Qu, Yongqiang Chen, Jun Li, Leng Liu

**Affiliations:** College of Chemistry and Chemical Engineering, Jinzhong University, Yuci 030619, China; quxy@jzxy.edu.cn (X.Q.); chenyongqiang@jzxy.edu.cn (Y.C.); hxx406@126.com (J.L.); liuleng@jzxy.edu.cn (L.L.)

**Keywords:** aryl thioamides, thiourea, C-H/C-N activation, C-S formation, transition-metal-free

## Abstract

Thiourea as a sulfur atom transfer reagent was applied for the synthesis of aryl thioamides through a three-component coupling reaction with aryl aldehydes and *N*,*N*-dimethylformamide (DMF) or *N*,*N*-dimethylacetamide (DMAC). The reaction could tolerate various functional groups and gave moderate to good yields of desired products under the transition-metal-free condition.

## 1. Introduction

The synthesis of sulfur-containing organic compounds has received much attention in recent years, due to their wide applications in biology, chemistry, and materials science [1,2,3,4,5,6,7,8,9,10]. There are many sulfur reagents for their synthesis, such as P_2_S_5_ [11], Lawesson’s reagent [12], disulfides [13,14,15], thiols [16,17,18,19], sulfonyl hydrazides [20,21,22,23], sodium sulfonate [24,25,26,27,28], and elemental sulfur [29,30,31,32]. Among them, both P_2_S_5_ and Lawesson’s reagent are the most widely used reagents, and yet they have an obvious drawback of being sensitive to moisture. Therefore, much better sulfur reagents have been pursued by the organic chemists for the past decades [33]. Thioamides, as an important class of sulfur-containing organic compounds, have been synthesized by applying different sulfur reagents as the sulfur source [1,34,35,36,37,38,39]. For example, Jiang et al. [35] reported that sodium sulfide as a sulfur source was applied for the synthesis of thioamides using aldehydes and *N*-substituted formamides. More recently, a coupling reaction between quaternary ammoniums, *N*-substituted formamides, and sodium disulfide was accomplished for rapid access to aryl thioamides [36]. Thiourea as an inexpensive and easy-to-handle sulfur atom transfer reagent, was used extensively as well, mainly for the synthesis of inorganic metal sulfides [40,41,42], organic thioethers [43,44,45,46,47], and thioesters [48,49]. As far as we known, a similar reaction using thiourea and aldehydes to prepare thioamides has not yet been introduced. Hence, we want to report a new three-component coupling reaction between aryl aldehydes, thiourea as an effective sulfur source, and DMF or DMAC, for the synthesis of various aryl thioamides.

## 2. Results and Discussion 

Initially, we treated the reaction of 4-chlorobenzaldehyde **1a** in DMF and H_2_O at 125 °C in the presence of thiourea using the benzoyl peroxide (BPO) as an oxidant. After 24 h, the desired thioamide product **3a** was isolated in 58% yield (Table 1, Entry 1). Subsequently, various oxidants, which are commonly used in C-H activation, such as *p*-benzoquinone (BQ), di-*t*-butyl peroxide (DTBP), *tert*-butyl hydroperoxide (TBHP), K_2_S_2_O_8_, or (NH_4_)_2_S_2_O_8_, were attempted, to optimize the reaction condition (Table 1, Entries 2–6). Among them, K_2_S_2_O_8_ proved to be best to give the desired thioamide product **3a** in 69% yield (Table 1, Entry 5). For this transformation, H_2_O played an extremely important role. No desired product **3a** was observed when increasing the concentration of H_2_O to 42 M or without addition of H_2_O (Table 1, Entries 7 and 9). Slightly enhancing or reducing the loading amount of K_2_S_2_O_8_, the yield of **3a** was not obviously changeable (Table 1, Entries 10–11). When the 20% of Cu(OAc)_2_ were used as a catalyst, only 54% yield of **3a** was afforded (Table 1, Entry 12) [36]. To our delight, the yield of **3a** was further promoted to 80% when 5 equiv. of pyridine (Py) as an additive were added (Table 1, Entry 13) [35]. 

**Scheme 1:**
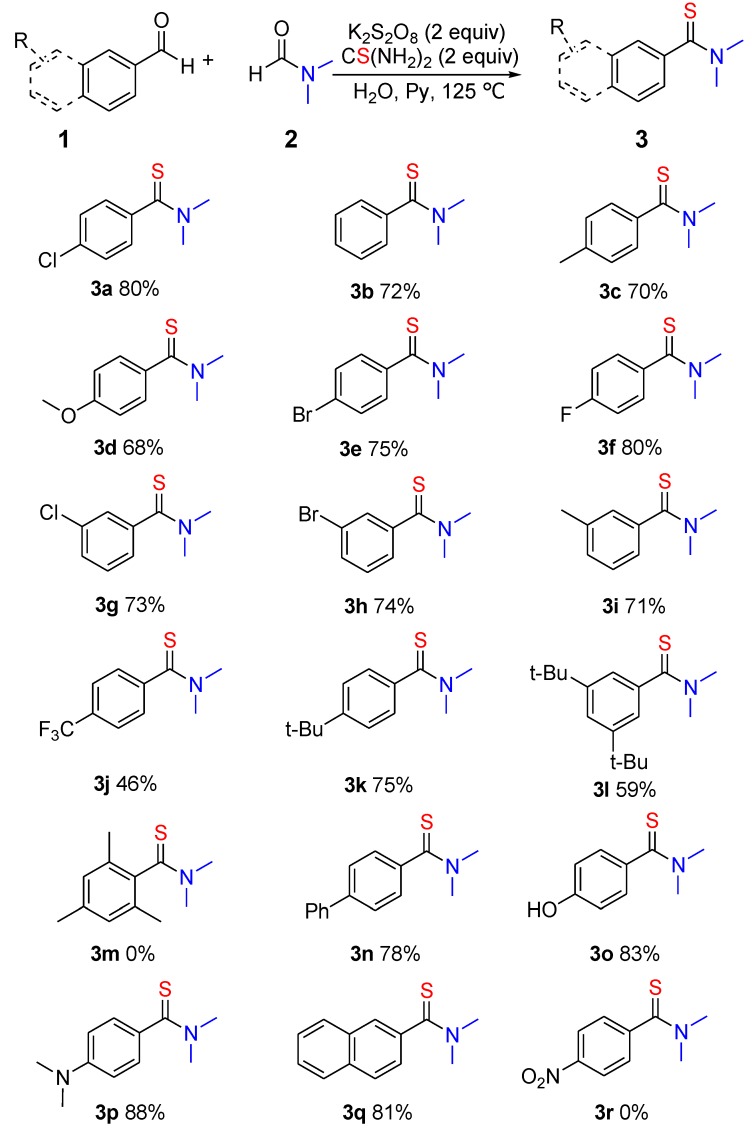
The substrate scope of substituted benzaldehydes. Reaction condition: aryl aldehyde **1** (0.25 mmol), thiourea (0.5 mmol), H_2_O (14 M), K_2_S_2_O_8_ (0.5 mmol) and Py (5 equiv.) in DMF (1.5 mL) at 125 °C for 24 h in sealed tube. Isolated yields were given.

After establishing the optimized conditions, this procedure was applied to access a variety of aryl thioamide derivatives. Several different aryl aldehydes could undergo this transformation smoothly in a mild condition to give the desired products **3a**–**r** (Scheme 1). The results indicated that many popular functional groups were well tolerated, such as methyl, methoxyl, chloro, bromo, fluoro, trifluoromethyl, and *tert*-butyl. Furthermore, the substrate bearing a sensitive hydroxy group, which was generally protected in the presence of an oxidant could be also tolerated in this transformation, and afforded the desired product **3o** in 83% yield. A substituted amino was suitable as well, and gave 88% yield of **3p**. The substituents on aromatic aldehydes had a certain influence on this transformation. When the substituents were strong electron-withdrawing groups, either lower yield of desired products, or no desired products were obtained (**3j** and **3r**). The desired product **3m** was not afforded possibly due to the steric hindrance. Moreover, our experiments demonstrated that 2-naphthaldehyde was a suitable substrate for this transformation, and gave the desired product **3q** a good yield.

**Scheme 2:**
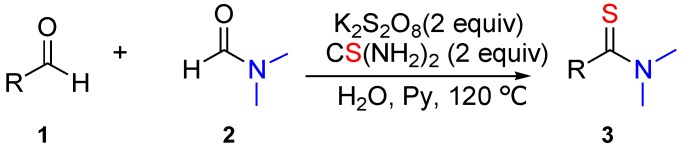

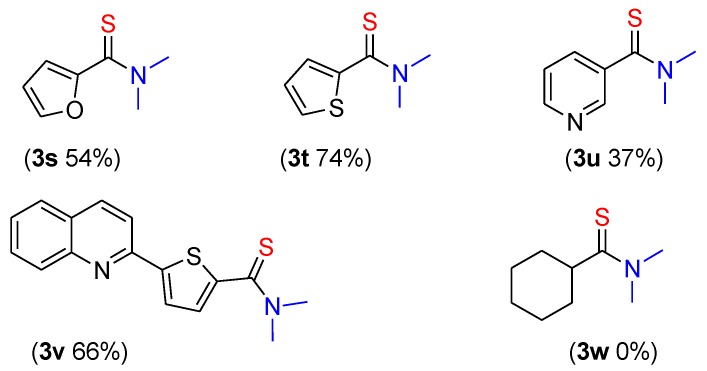
The substrate scope of other aldehydes. Reaction condition: aldehyde **1** (0.25 mmol), thiourea (0.5 mmol), H_2_O (14 M), K_2_S_2_O_8_ (0.5 mmol), and Py (5 equiv.) in DMF (1.5 mL) at 125 °C for 24 h in sealed tube. Isolated yields were given.

To further expand the substrate scope, some selected heterocyclic aldehydes and an aliphatic aldehyde were examined under the optimal condition (Scheme 2). Generally, five or six members heterocyclic derivatives were suitable for this transformation, such as furan-2-carbaldehyde, thiophene-2-carbaldehyde, and nicotinaldehyde, giving the corresponding thioamide products **3s**, **3t**, and **3u** 54%, 74%, and 37% yields, respectively. In addition, aliphatic aldehyde did not accomplish this transformation (**3w**).

We attempted to explore the different *N*-substituted formamides for this transformation in additional solvent. No good results were provided when the normal solvents such as *N-*methyl-2-pyrrolidone (NMP), 1,4-dioxane, 1,2-dichloroethane (DCE), toluene, chlorobenzene (PhCl), dimethyl sulfoxide (DMSO), ethylene glycol, were used (see Supporting Information). Unexpectedly, *N*,*N*-dimethylacetamide (DMAC), which was seldom used as an amine source by the acyl C-N bond activation [37], could replace DMF to give the same desired product in good yield. Subsequently, the reactions of aryl aldehydes with thiourea in DMAC were examined under the similar reaction condition (Table 2). The results demonstrated that various groups were tolerated well, such as methyl, chloro, and bromo, and the good yields of the desired products were isolated.

In addition, extremely small amounts of amide products were observed, along with the generation of thioamide products under the optimal condition. So, two control experiments were conducted to explain the tentative reaction mechanism (see Supporting Information). First, no thioamide product was formed in the absence of thiourea, and only trace amounts of amide product were observed. Second, when the *N*,*N*-dimethylbenzamide replacing the benzaldehyde was manipulated under the standard condition, no desired thioamide was observed. 

**Scheme 3:**
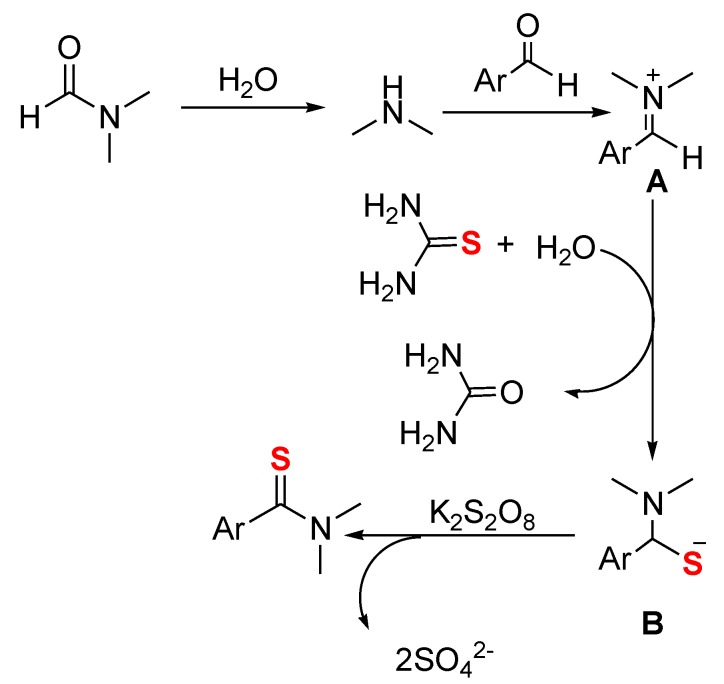
Plausible reaction pathway.

Based on our experimental results and previous reports [35], a proposed reaction mechanism for this transformation is described in Scheme 3. First, an aryl aldehyde undergoes a nucleophilic attack by a dimethylamine, which is from the hydrolysis of DMF, to generate iminium intermediate **A**. The iminium **A** then is directly attacked by thiourea to form the intermediate **B**, together with the release of urea [43,47,49]. Finally, intermediate **B** is oxidized by K_2_S_2_O_8_ to afford the desired thioamide product.

## 3. Materials and Methods 

Unless otherwise stated, all reagents and solvents were purchased from commercial suppliers, and were used without further purification. Reactions were monitored by thin layer chromatography (TLC) analysis on silica gel 60 F254, and visualization was accomplished by irradiation with short wave UV light at 254 nm. ^1^H-NMR and ^13^C-NMR spectra were recorded on a Bruker Avance 400 or a 500 MHz spectrometer (Bruker, Karlsruhe, Germany), with tetramethylsilane (TMS) as the internal standard. The coupling constants *J* are given in Hz. Mass spectra were measured with the Thermo Scientific LTQ Orbitrap XL MS spectrometer (Thermo Fisher Scientific, Waltham, MA, USA) or GC-MS QP2010 (Comfort Technology Limited, Kowloon, Hong Kong).

A mixture of aldehyde **1** (0.25 mmol), thiourea (0.5 mmol), K_2_S_2_O_8_ (0.5 mmol), and Py (1.25 mmol) in 2.0 mL DMF/H_2_O (*v*/*v* = 3:1) was stirred in a sealed tube under air at 125 °C for 24 h. After the reaction was achieved, the crude mixture was purified by column chromatography (silica gel, EtOAc/petroleum ether) to afford the desired product **3**.

*4-Chloro-N,N-dimethylbenzothioamide* (**3a**) [35]. ^1^H-NMR (CDCl_3_, 400 MHz): δ (ppm): 7.36–7.33 (m, 2H), 7.29–7.26 (m, 2H), 3.61 (s, 3H), 3.19 (s, 3H); ^13^C-NMR (CDCl_3_, 100 MHz): δ (ppm): 199.91, 141.66, 134.60, 128.58, 127.29, 44.18, 43.32; HRMS (ESI) *m*/*z* calculated (calcd.) for C_9_H_11_ClNS^+^ (M + H)^+^ 200.02952, found 200.02971.

*N,N-Dimethylbenzothioamide* (**3b**) [35]. ^1^H-NMR (CDCl_3_, 400 MHz): δ (ppm): 7.37–7.30 (m, 5H), 3.62 (s, 3H), 3.19 (s, 3H); ^13^C-NMR (CDCl_3_, 100 MHz): δ (ppm): 201.65, 143.69, 128.89, 128.64, 126.04, 44.44, 43.53; GC-MS (EI) *m*/*z* (%) 165.10 (70, M^+^), 164.05 (98), 121.05 (100), 77.00 (46).

*4-Methyl-N,N-dimethylbenzothioamide***3c** [35]. ^1^H-NMR (CDCl_3_, 400 MHz): δ (ppm): 7.23 (d, *J* = 8 Hz, 2H), 7.16 (d, *J* = 8 Hz, 2H), 3.61 (s, 3H), 3.20 (s, 3H), 2.36 (s, 3H); ^13^C-NMR (CDCl_3_, 100 MHz): δ (ppm): 201.55, 140.59, 138.68, 128.89, 125.89, 44.22, 43.35, 21.27; GC-MS (EI) *m*/*z* (%) 179.05 (80, M^+^). 178.05 (100), 145.10 (50), 135.05 (98), 91.05 (45).

*4-Methoxy-N,N-dimethylbenzothioamide* (**3d**) [35]. ^1^H-NMR (CDCl_3_, 400 MHz): δ (ppm): 7.31 (d, *J* = 12 Hz, 2H), 6.87 (d, *J* = 12 Hz, 2H), 3.82 (s, 3H), 3.59 (s, 3H), 3.22 (s, 3H); ^13^C-NMR (CDCl_3_, 100 MHz): δ (ppm): 201.31, 160.02, 135.82, 127.90, 113.51, 55.40, 44.37, 43.59; GC-MS (EI) *m*/*z* (%) 195.05 (M^+^, 84), 194.05 (93), 151.05 (100).

*4-Bromo-N,N-dimethylbenzothioamide* (**3e**) [35]. ^1^H-NMR (CDCl_3_, 400 MHz): δ (ppm): 7.53–7.50 (m, 2H), 7.23–7.19 (m, 2H), 3.60 (s, 3H), 3.19 (s, 3H); ^13^C-NMR (CDCl_3_, 100 MHz): δ (ppm): 199.79, 142.12, 131.53, 127.52, 122.73, 44.20, 44.30; GC-MS (EI) *m*/*z* (%) 242.95 (63, M^+^), 243.90 (100), 242.95 (63), 241.90 (92), 200.85 (47), 198.90 (48), 120.00 (54).

*4-Fluoro-N,N-dimethylbenzothioamide* (**3f**) [35]. ^1^H-NMR (CDCl_3_, 400 MHz): δ (ppm): 7.32–7.28 (m, 2 H), 7.05–6.99 (m, 2H), 3.58 (s, 3H), 3.16 (s, 3H); ^13^C-NMR (CDCl_3_, 100 MHz): δ (ppm): 200.19, 162.66 (d, *J =* 248 Hz), 139.44 (d, *J =* 4 Hz), 128.00 (d, *J =* 9 Hz), 115.33 (d, *J =* 22 Hz), 44.24, 43.45.

*3-Chloro-N,N-dimethylbenzothioamide* (**3g**) [35]. ^1^H-NMR (CDCl_3_, 400 MHz): δ (ppm): 7.33–7.25 (m, 3H), 7.20–7.18 (m, 1H), 3.60 (s, 3H), 3.19 (s, 3H); ^13^C-NMR (CDCl_3_, 100 MHz): δ (ppm): 199.11, 144.78, 134.29, 129.76, 128.63, 125.89, 123.83, 44.18, 43.18; GC-MS (EI) *m*/*z* (%) 200.00 (43), 199.0 (69, M^+^). 198.00 (100), 165.00 (32), 157.00 (26), 155.00 (74), 111.00 (24).

*3-Bromo-N,N-dimethylbenzothioamide* (**3h**) [35]. ^1^H-NMR (CDCl_3_, 400 MHz): δ (ppm): 7.50–7.46 (m, 2H), 7.27–7.22 (m, 2H), 3.60 (s, 3H), 3.19 (s, 3H); ^13^C-NMR (CDCl_3_, 100 MHz): δ (ppm): 199.01, 144.98, 131.56, 129.99, 128.68, 124.30, 122.38, 44.19, 43.19; GC-MS (EI) *m*/*z* (%) 244.95 (64), 243.95 (100), 242.95 (65, M^+^), 241.95 (95), 200.90 (43), 198.90 (43), 120.00 (51).

*3-Methyl-N,N-dimethylbenzothioamide* (**3i**) [35]. ^1^H-NMR (CDCl_3_, 400 MHz): δ (ppm): 7.34–7.24 (m, 1H), 7.17 (d, *J* = 6.0 Hz, 2H), 7.11 (d, *J* = 7.5 Hz, 1H), 3.64 (s, 3H), 3.21 (s, 3H), 2.40 (s, 3H); ^13^C-NMR (CDCl_3_, 100 MHz): δ (ppm): 201.2, 143.2, 138.0, 129.1, 128.0, 126.1, 122.4, 44.0, 43.0, 21.2.

*4-(Trifluoromethyl)-N,N-dimethylbenzothioamide* (**3j**) [35]. ^1^H-NMR (CDCl_3_, 400 MHz): δ (ppm): 7.64 (d, *J* = 8 Hz, 2H), 7.43 (d, *J* = 12 Hz, 2H), 3.63 (s, 3H), 3.18 (s, 3H); ^13^C-NMR (CDCl_3_, 100 MHz): δ (ppm): 199.30, 146.56,130.46 (q, *J =* 32.6 Hz),126.02, 125.56 (q, *J =* 3.8 Hz), 123.73 (q, *J =* 270.5 Hz), 44.07, 43.09; GC-MS (EI) *m*/*z* (%) 233.00 (M^+^, 71), 232.00 (100), 199.05 (37), 189.00 (64).

*4-tert-Butyl-N,N-dimethylbenzothioamide* (**3k**) [37]. ^1^H-NMR (CDCl_3_, 400 MHz): δ (ppm): 7.38–7.35 (m, 2H), 7.28–7.24 (m, 2H), 3.61 (s, 3H), 3.21 (s, 3H), 1.32 (s, 9H); ^13^C-NMR (CDCl_3_, 100 MHz): δ (ppm): 201.64, 151.78, 140.49, 125.67, 125.22, 44.27, 43.34, 34.70, 31.23. GC-MS (EI) *m*/*z* (%) 221.10 (70, M^+^), 220.05 (70), 147.05 (24).

*3,5-di-tert-Butyl-N,N-dimethylbenzothioamide* (**3l**). ^1^H-NMR (CDCl_3_, 400 MHz): δ (ppm): 7.39 (t, *J* = 4 Hz, 1H), 7.15 (d, *J* = 4 Hz, 2H), 3.63 (s, 3H), 3.16 (s, 3H), 1.33 (s, 18H); ^13^C-NMR (CDCl_3_, 100 MHz): δ (ppm): 202.78, 150.70, 142.63, 122.75, 120.13, 44.21, 43.32, 34.92, 31.40. GC-MS (EI) *m*/*z* (%) 277.15 (86, M^+^), 276.15 (100), 220.05 (80). HRMS (ESI) *m*/*z* calcd. for C_17_H_28_NS^+^ (M + H)^+^ 278.19370, found 278.19366.

*4-Benzyl-N,N-dimethylbenzothioamide* (**3n**) [38]. ^1^H-NMR (CDCl_3_, 400 MHz): δ (ppm): 7.59–7.56 (m, 4H), 7.47–7.43 (m, 2H), 7.41–7.34 (m, 3H),3.62 (s, 3H), 3.23 (s, 3H); ^13^C-NMR (CDCl_3_, 100 MHz): δ (ppm): 201.04, 142.17, 141.56, 140.33, 128.89, 127.69, 127.10, 127.09, 126.39, 44.28, 43.32. GC-MS (EI) *m*/*z* (%) 241.05 (78, M^+^), 240.05 (100), 197.00 (58), 181.05 (66), 152.05 (74).

*4-Hydroxy-N,N-dimethylbenzothioamide* (**3o**) [35]. ^1^H-NMR (CDCl_3_, 400 MHz): δ (ppm): 7.21–7.15 (m, 2H), 6.75–6.70 (m, 2H), 5.89 (br s, 1H), 3.59 (s, 3H), 3.21 (s, 3H); ^13^C-NMR (CDCl_3_, 100 MHz): δ (ppm): 200.97, 156. 03, 136. 00, 127.45, 114.85, 43.97, 43.20; GC-MS (EI) *m*/*z* (%) 181.05 (M^+^, 74), 180.05 (79), 147.10 (31), 137.05 (100).

*4-(Dimethylamino)-N,N-dimethylbenzothioamide* (**3p**) [50]. ^1^H-NMR (CDCl_3_, 400 MHz): δ (ppm): 7.32 (d, *J* = 8 Hz, 2H), 7.63 (d, *J* = 8 Hz, 2H), 3.59 (s, 3H), 3.28 (s, 3H), 2.99 (s, 6H); ^13^C-NMR (CDCl_3_, 100 MHz): δ (ppm): 201.64, 150.54, 130.30, 127.98, 110.56, 44.19, 43.40, 39.86; GC-MS (EI) *m*/*z* (%) 208.05 (M^+^, 74), 207.05 (46), 164.05 (100), 148.10 (57).

*N,N-Dimethylnaphthalene-2-carbothioamide* (**3q**) [35]. ^1^H-NMR (CDCl_3_, 400 MHz): δ (ppm): 7.82–7.81 (m, 3H), 7.77 (s, 1H), 7.53–7.46 (m, 2H), 7.44–7.41(m, 1H), 3.64 (s, 3H), 3.19 (s, 3H); ^13^C-NMR (CDCl_3_, 100 MHz): δ (ppm): 201.21, 140.60, 128.38, 128.20, 127.75, 126.78, 126.74, 124.73, 123.94, 44.27, 43.31.

*N,N-Dimethylfuran-2-carbothioamide* (**3s**) [51]. ^1^H-NMR (CDCl_3_, 400 MHz): δ (ppm): 7.47 (d, *J* = 4 Hz, 1H), 7.09 (d, *J* = 4 Hz, 1H), 6.46 (d, *J* = 4 Hz, 1H),3.56 (s, 3H), 3.45 (s, 3H); ^13^C-NMR (CDCl_3_, 100 MHz): δ (ppm): 158.31, 151.98, 142.80, 117.27, 111.49, 44.01, 43.80. GC-MS (EI) *m*/*z* (%) 155.05 (100, M^+^), 111.00 (70), 73.95 (27).

*N,N-Dimethylthiophene-2-carbothioamide* (**3t**) [35]. ^1^H-NMR (CDCl_3_, 400 MHz): δ (ppm): 7.41 (dd, *J* = 4.8, 0.8 Hz, 1H), 7.12 (dd, *J* = 4.0, 1.2 Hz, 1H), 6.99–6.97 (m, 1H), 3.58 (s, 3H), 3.45 (s, 3H); ^13^C-NMR (CDCl_3_, 100 MHz): δ (ppm):191.63, 145.23, 129.30, 126.50, 126.45, 44.65. GC-MS (EI) *m*/*z* (%) 171.00 (77, M^+^), 127.00 (100).

*N,N-Dimethylpyridine-3-carbothioamide* (**3u**) [52]. ^1^H-NMR (CDCl_3_, 400 MHz): δ (ppm): 8.59–8.57 (m, 2H),7.69 (dt, *J* = 8.0, 2.0 Hz, 1H), 7.33–7.30 (m, 1H), 3.63 (s, 3H), 3.23 (s, 3H); ^13^C-NMR (CDCl_3_, 100 MHz): δ (ppm):197.59, 149.63, 146.19, 133.70, 123.18, 44.25, 43.37. GC-MS (EI) *m*/*z* (%) 166.05 (85, M^+^), 165.05 (88), 149.10 (35), 122.00 (100), 106.05 (38), 78.00 (62).

*N,N-Dimethyl-5-(quinolin-2-yl) thiophene-2-carbothioamide* (**3v**). ^1^H-NMR (CDCl_3_, 400 MHz): δ (ppm): 8.15 (d, *J* = 8 Hz, 1H), 8.08 (d, *J* = 8 Hz, 1H), 7.78 (t, *J* = 8 Hz, 2H), 7.74–7.69 (m, 1H), 7.59 (d, *J* = 4 Hz, 1H), 7.54–7.49 (m, 1H), 7.19 (d, *J* = 4 Hz, 1H), 3.56 (d, *J* = 40 Hz, 6H); ^13^C-NMR (CDCl_3_, 100 MHz): δ (ppm): 191.56, 151.54, 148.18, 148.08, 146.86, 136.77, 130.01, 129.37, 127.82, 127.53, 127.40, 126.49, 124.94, 117.42, 44.54. HRMS (ESI) *m*/*z* calculated (calcd.) for C_16_H_15_N_2_S_2_^+^ (M + H)^+^ 299.06712, found 299.06706.

## 4. Conclusions

In conclusion, we have demonstrated an efficient and transitional-metal-free method for the synthesis of aryl thioamides derived from aryl aldehydes using thiourea as a sulfur source in the presence of potassium persulfate, in DMF or DMAC. This strategy has the advantages of good functional-group tolerance and gives moderate to good yields of desired products. Further studies on synthetic applications are currently under way.

## Figures and Tables

**Table 1:** Optimization of reaction conditions ^a^. 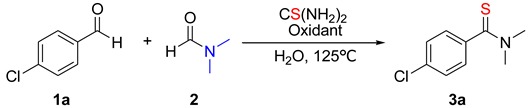

Entry	Oxidant (Equiv)	Concentration of H_2_O (M)	Yield (%) ^b^
1	BPO (2)	14	58
2	BQ (2)	14	0
3	DTBP (2)	14	<5
4	TBHP (2)	14	20
5	K_2_S_2_O_8_ (2)	14	69
6	(NH_4_)_2_S_2_O_8_ (2)	14	55
7	K_2_S_2_O_8_ (2)	42	0 ^c^
8	K_2_S_2_O_8_ (2)	8	65 ^d^
9	K_2_S_2_O_8_ (2)	0	0 ^e^
10	K_2_S_2_O_8_ (3)	14	68
11	K_2_S_2_O_8_ (1.8)	14	61
12	K_2_S_2_O_8_ (2)	14	54 ^f^
13	K_2_S_2_O_8_ (2)	14	80 ^g^

^a^ Condition: **1a** (0.25 mmol), **2** (9.6 M), thiourea (0.5 mmol), oxidant, H_2_O, 125 °C, 24 h. ^b^ Isolated yield. ^c^ 3.4 M of DMF were used. ^d^ 11 M of DMF were used. ^e^ 13 M of DMF were used. ^f^ 20 mol% of Cu(OAc)_2_ were added. ^g^ 5 equiv. of pyridine (Py) were added.

**Table 2:** The synthesis of aryl thioamides by DMAC ^a^. 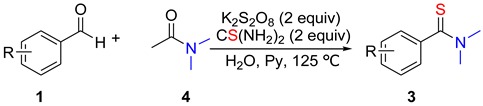

Entry	R	Yield (%) of 3 ^b^
1	4-Cl	63 (**3a**)
2	H	52 (**3b**)
3	4-CH_3_	55 (**3c**)
4	3-Br	48 (**3h**)
5	3-CH_3_	51 (**3i**)
6	4-Ph	60 (**3n**)

^a^ Condition: 1 (0.25 mmol), 4 (1.5 mL), thiourea (0.5 mmol), K_2_S_2_O_8_ (0.5 mmol), H_2_O (0.5 mL), Py (1.25 mmol), 125 °C, 36 h. ^b^ Isolated yield.

## Supplementary Materials

The following are available online at http://www.mdpi.com/1420-3049/23/9/2225/s1, Table S1: Screening of various solvents, Figure S1: Three control experiments for mechanism study, 1H-NMR, 13C-NMR, and MS spectrum of 3a–w.
