# Supplementary material for: K2S2O8-Promoted Aryl Thioamides Synthesis from Aryl Aldehydes Using Thiourea as the Sulfur Source
Source: Molecules. 2018 Sep 1;23(9):2225. doi: 10.3390/molecules23092225 (PMC6225268; doi:10.3390/molecules23092225)

# Electronic Supplementary Information (ESI)

# for *molecules*

# $K_2S_2O_8$-Promoted Aryl Thioamides Synthesis from Aryl Aldehydes Using Thiourea as the Sulfur Source

Yong-Jun Bian, * Xing-Yu Qu, Yong-qiang Chen, Jun Li, Leng Liu

*College of Chemistry and Chemical Engineering, Jinzhong University, Yuci 030619, P. R. China*

Email: yjbian2013@jzxy.edu.cn

# Part I. Experimental Section

## 1. **Table S1.** Screening of Various Solvents

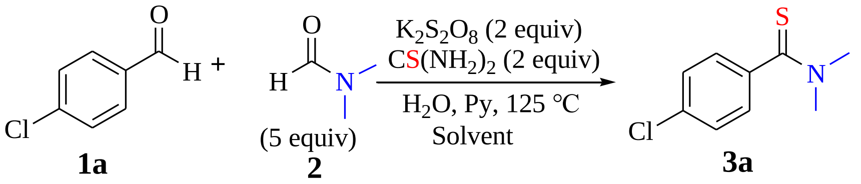


| Entry | Solvent | Yield (%)[b] |
|---|---|---|
| 1 | NMP | <5 |
| 2 | 1,4-dioxane | trace |
| 3 | DCE | trace |
| 4 | toluene | 0 |
| 5 | PhCl | 0 |
| 6 | DMSO | 0 |
| 7 | $HOCH_2CH_2OH$ | 0 |

[a] Conditions: **1a** (0.25 mmol), **2** (1.25 mmol), $K_2S_2O_8$ (0.5 mmol), thiourea (0.5 mmol), $H_2O$ (0.5 mL), Py (1.25 mmol), 125°C for 24 h in solvent (1.5 mL), unless otherwise noted. [b] Isolated yield.

## 2. **Three control experiments for mechanism study**.

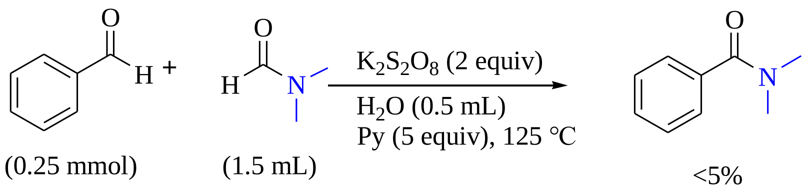


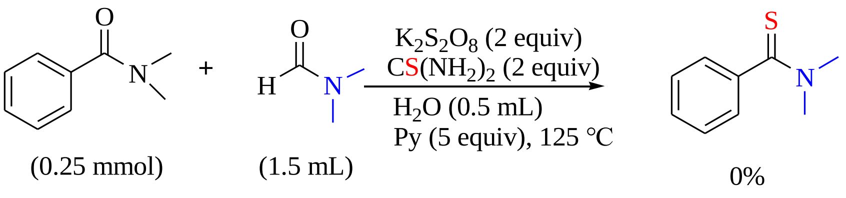

# **Part II** $^{1}$H NMR and $^{13}$C NMR

$^{1}$H NMR of 4-chloro-*N*, *N*-dimethylbenzothioamide **3a**

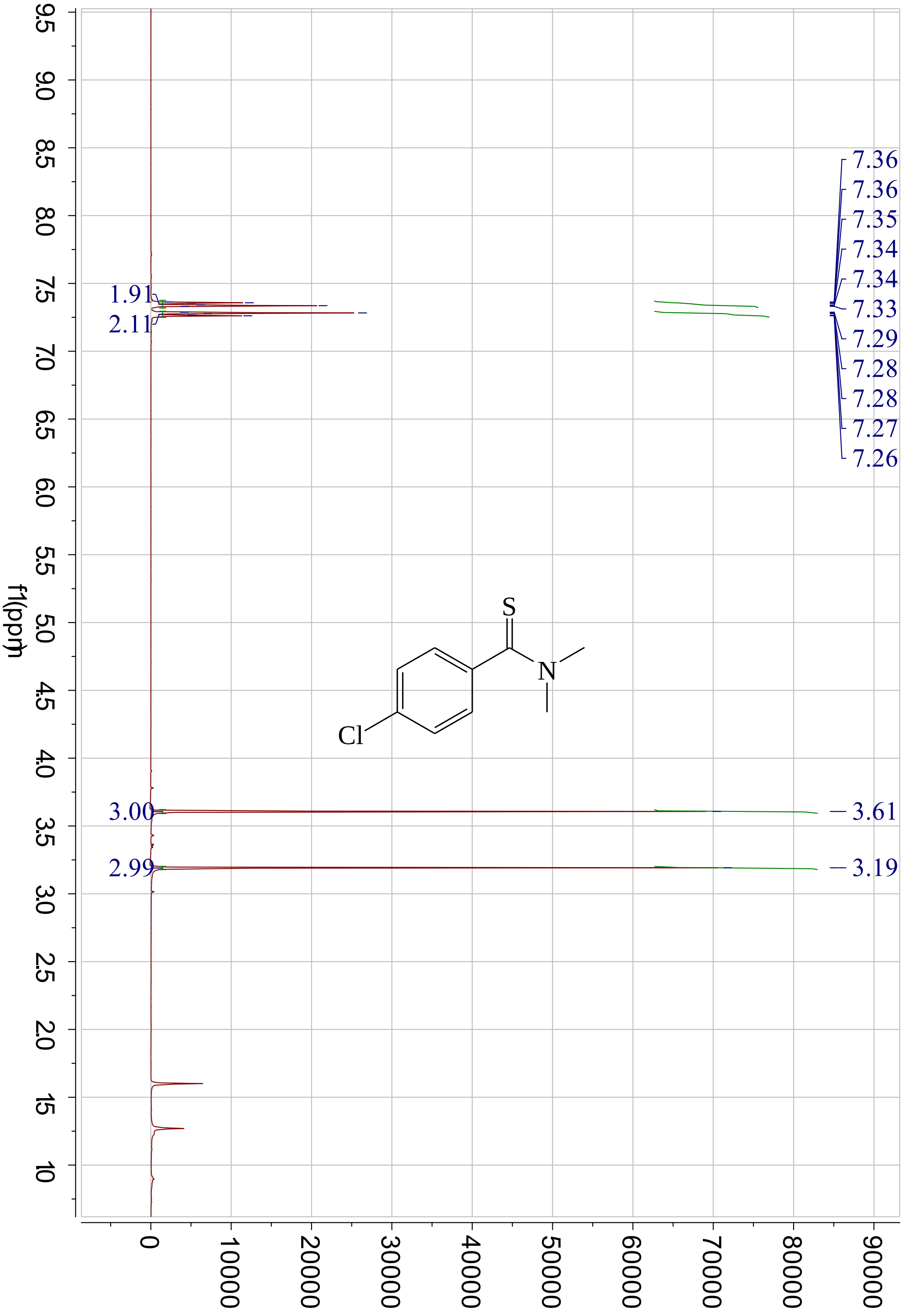

$^{1}$H NMR of *N*, *N*,4-trimethylbenzothioamide **3c**

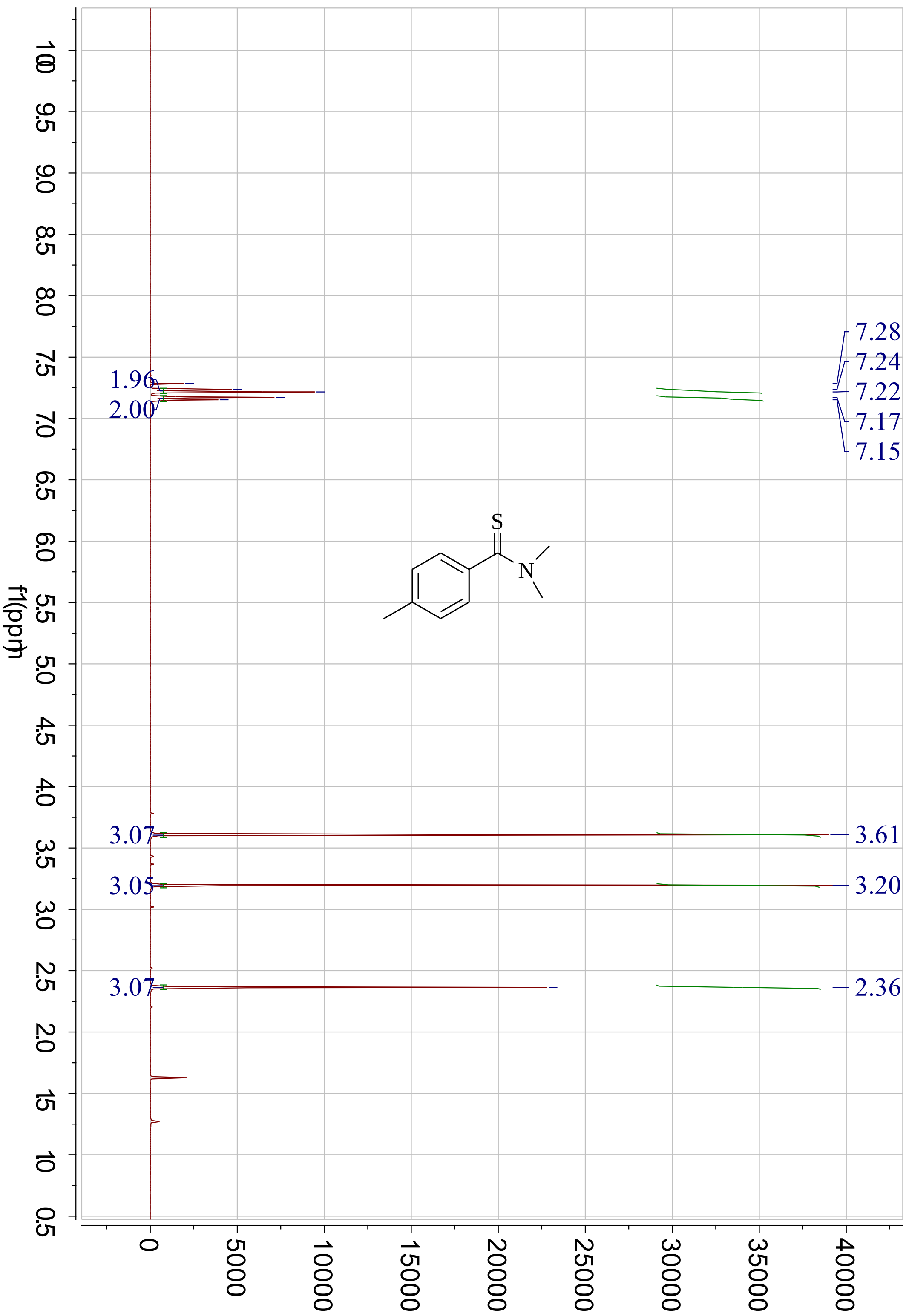

$^{1}$H NMR of 4-methoxy-*N*, *N*-dimethylbenzothioamide **3d**

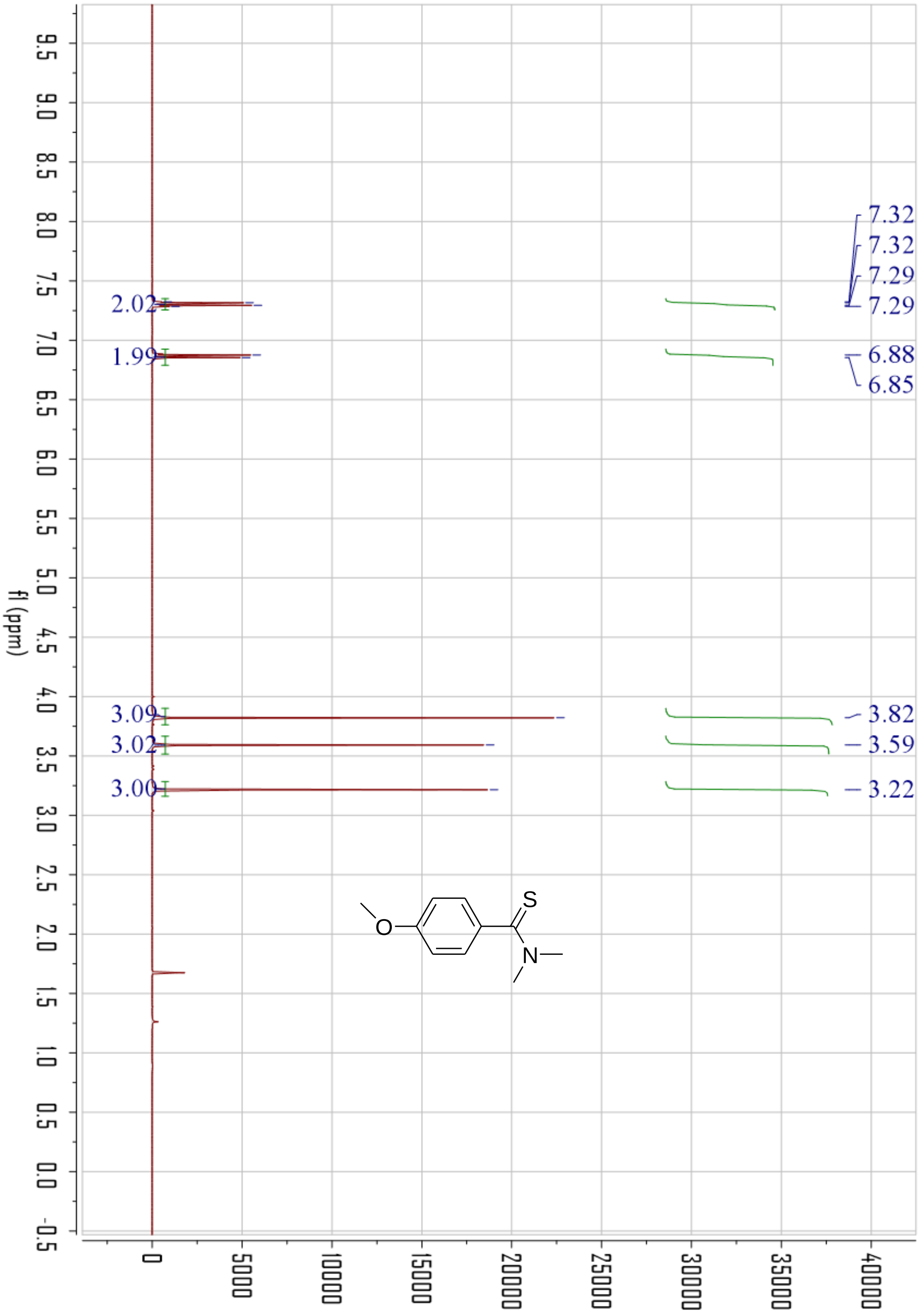

$^{13}$C NMR of 4-methoxy-*N*, *N*-dimethylbenzothioamide **3d**

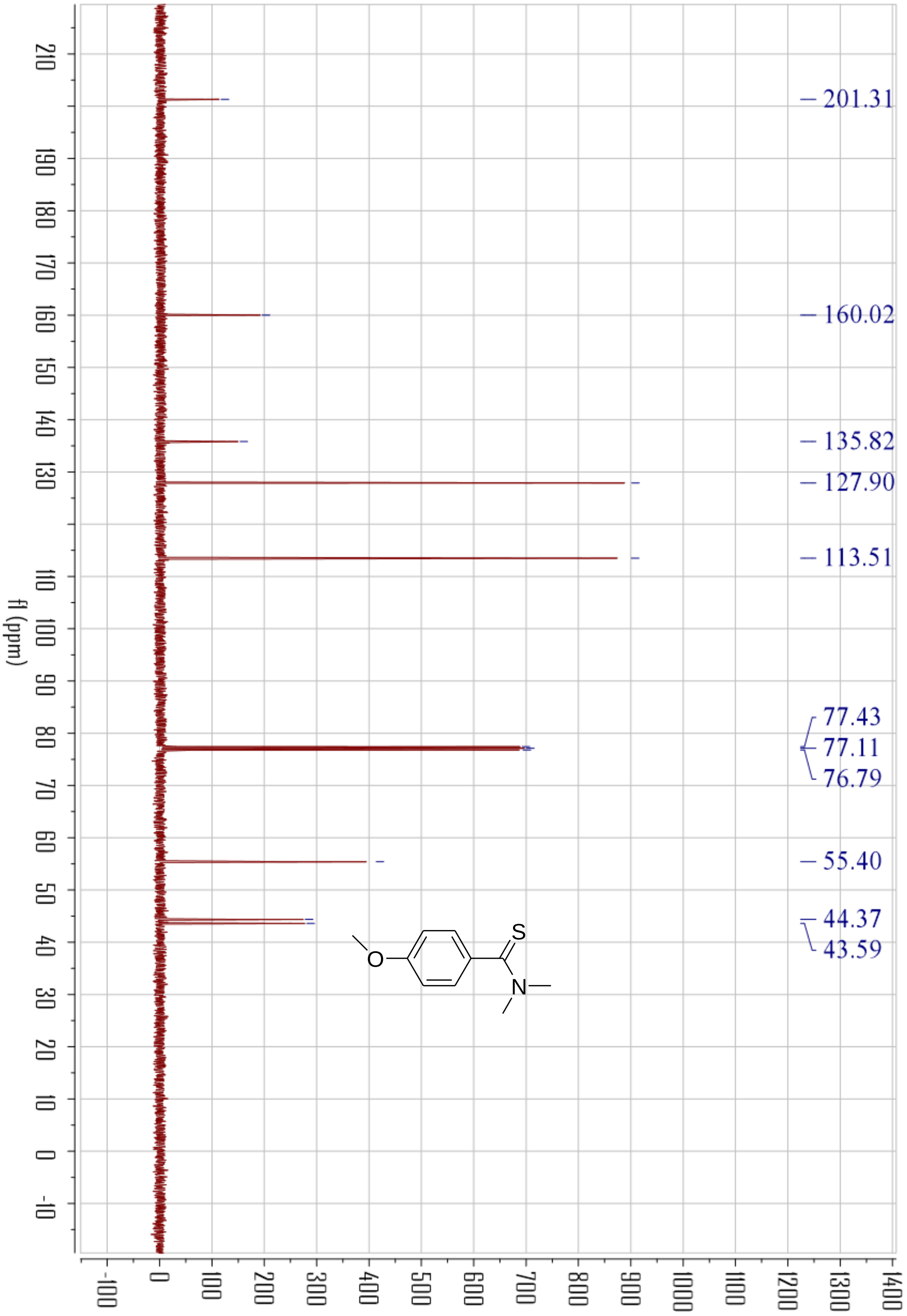

$^{1}$H NMR of 4-bromo-*N*, *N*-dimethylbenzothioamide **3e**

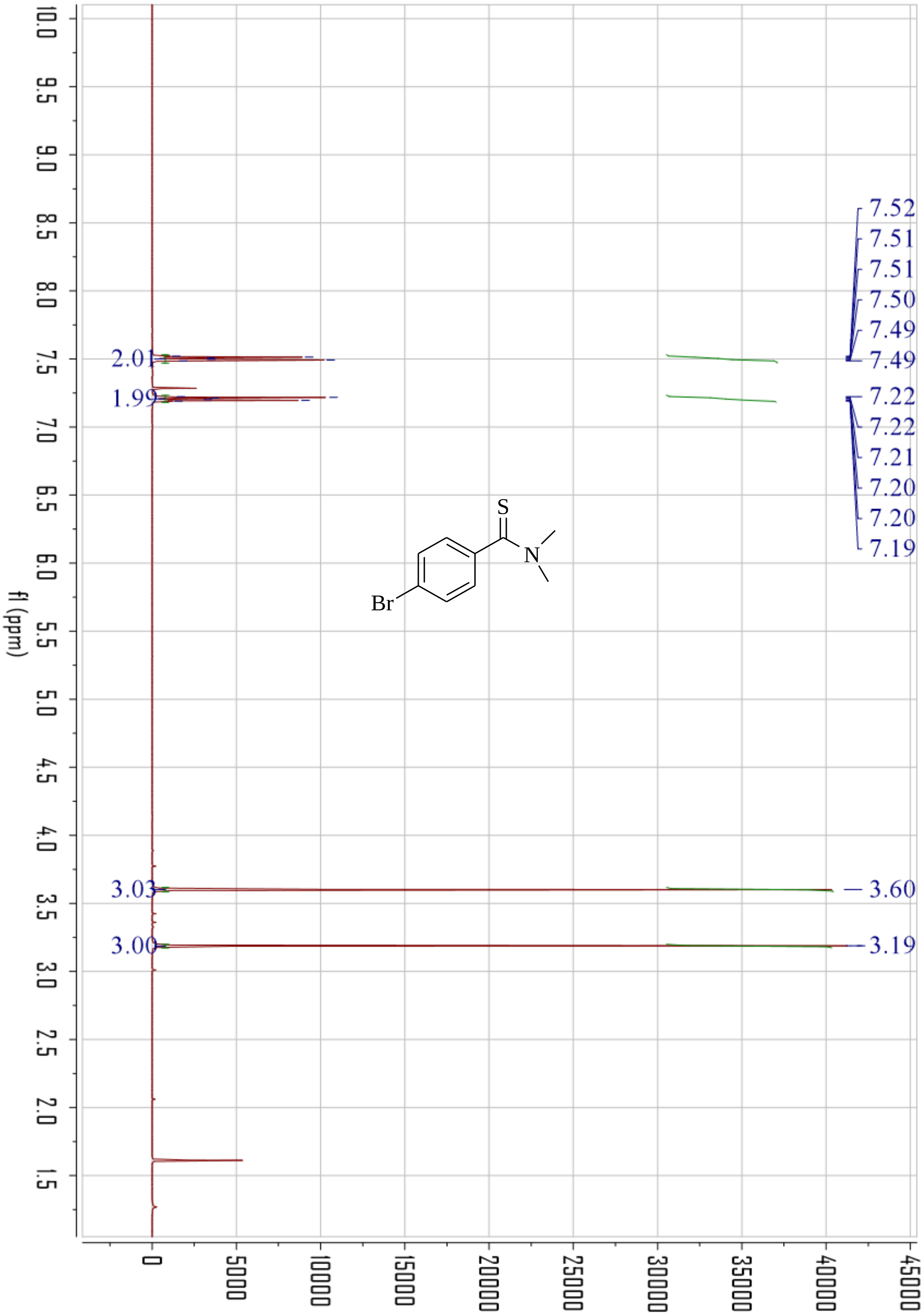

$^{1}$H NMR of 4-fluoro-*N*, *N*-dimethylbenzothioamide **3f**

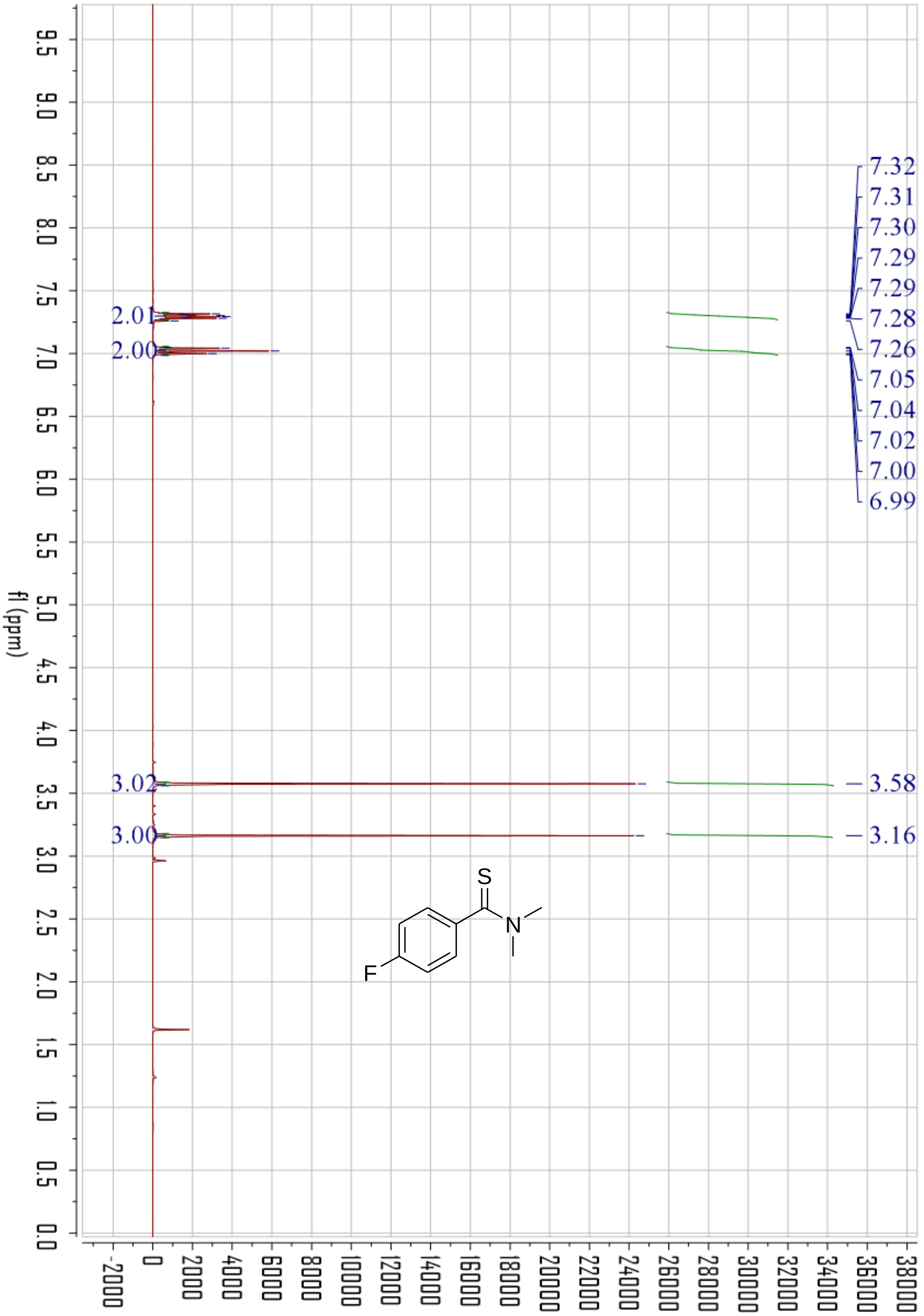

$^{13}$C NMR of 4-fluoro-*N*, *N*-dimethylbenzothioamide **3f**

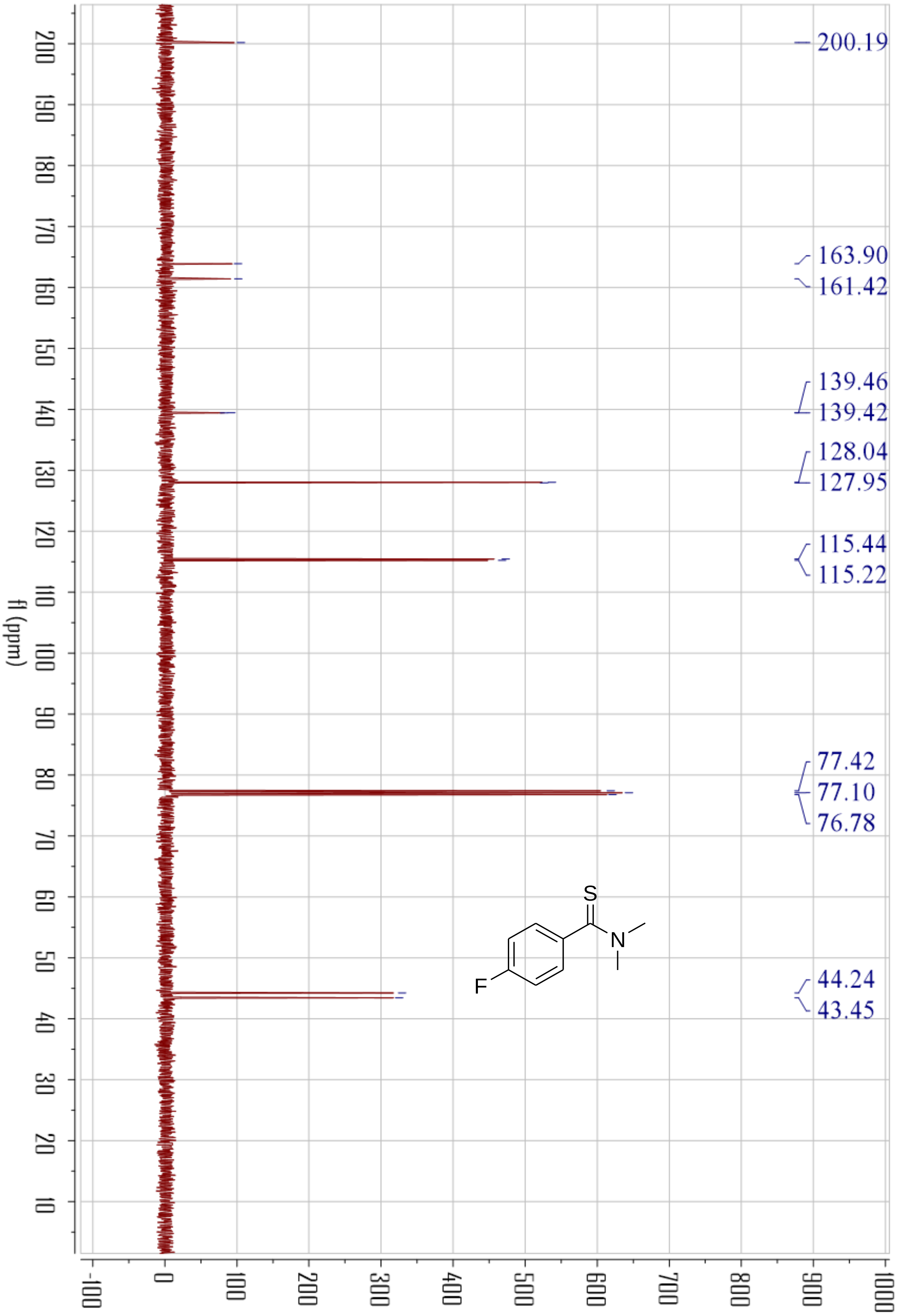

$^{1}$H NMR of 3-chloro-*N*, *N*-dimethylbenzothioamide **3g**

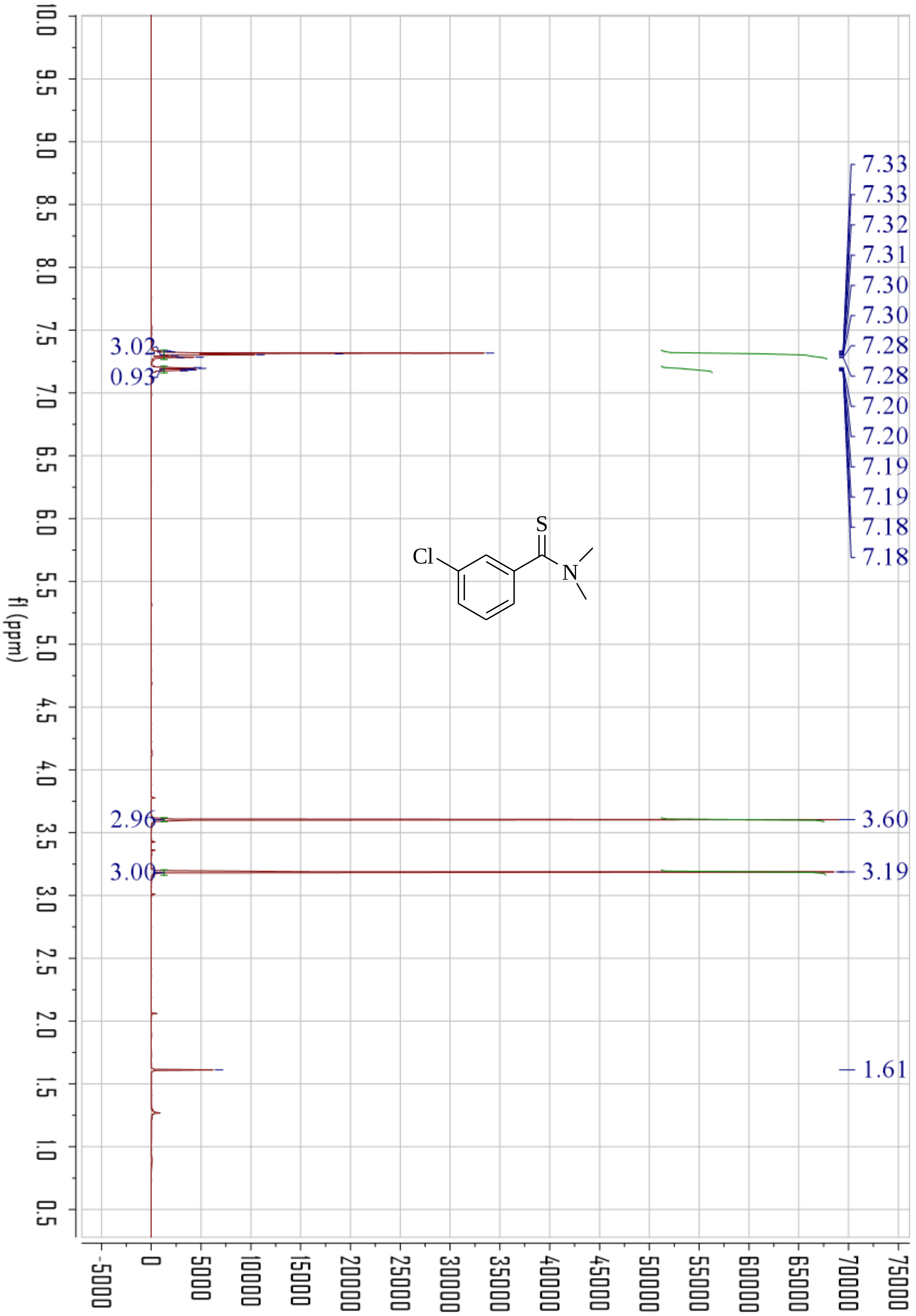

$^{1}$H NMR of 4-(trifluoromethyl)-*N*, *N*-dimethylbenzothioamide **3j**

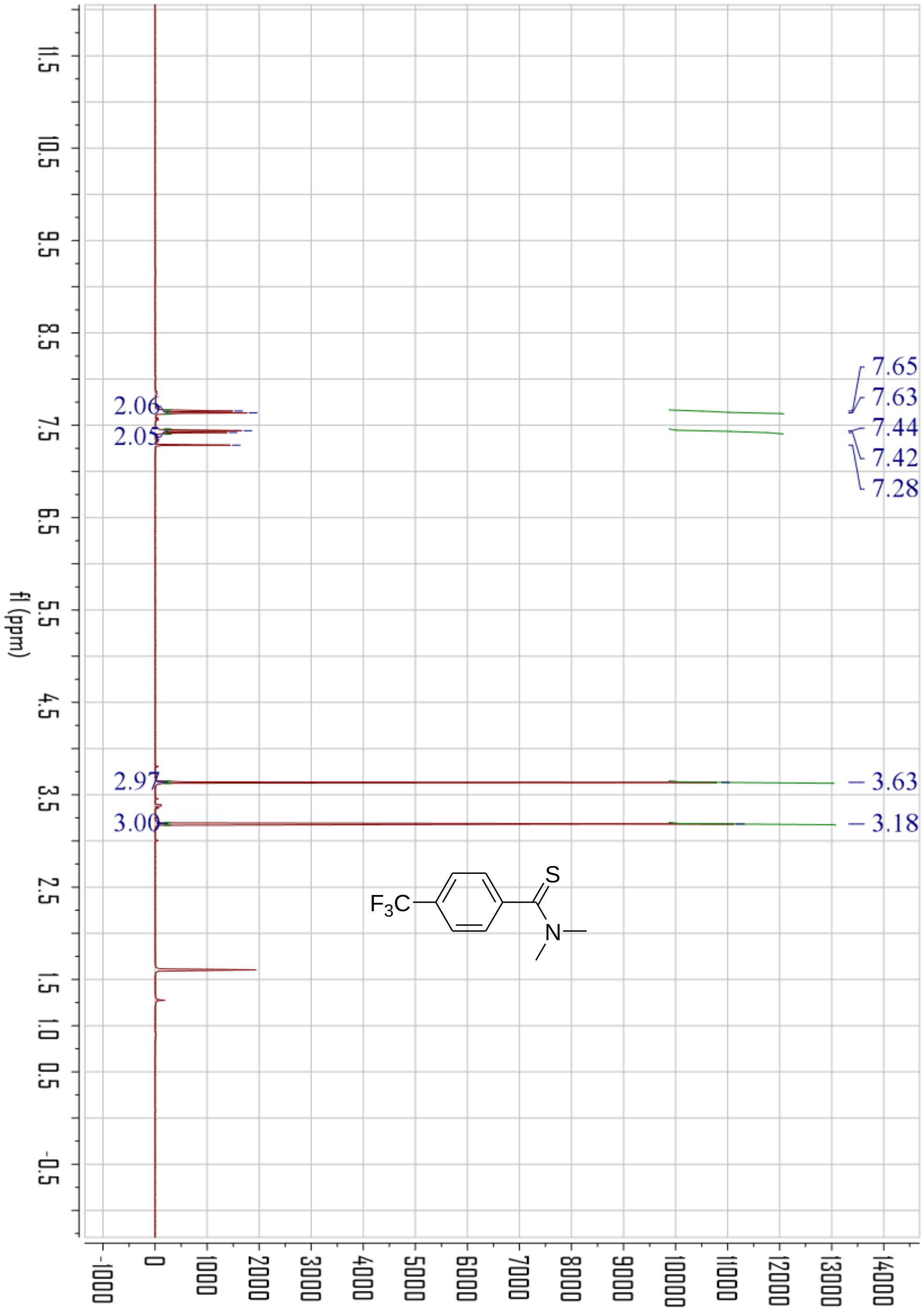

$^{1}$H NMR of 4-tert-butyl-*N*, *N*-dimethylbenzothioamide **3k**

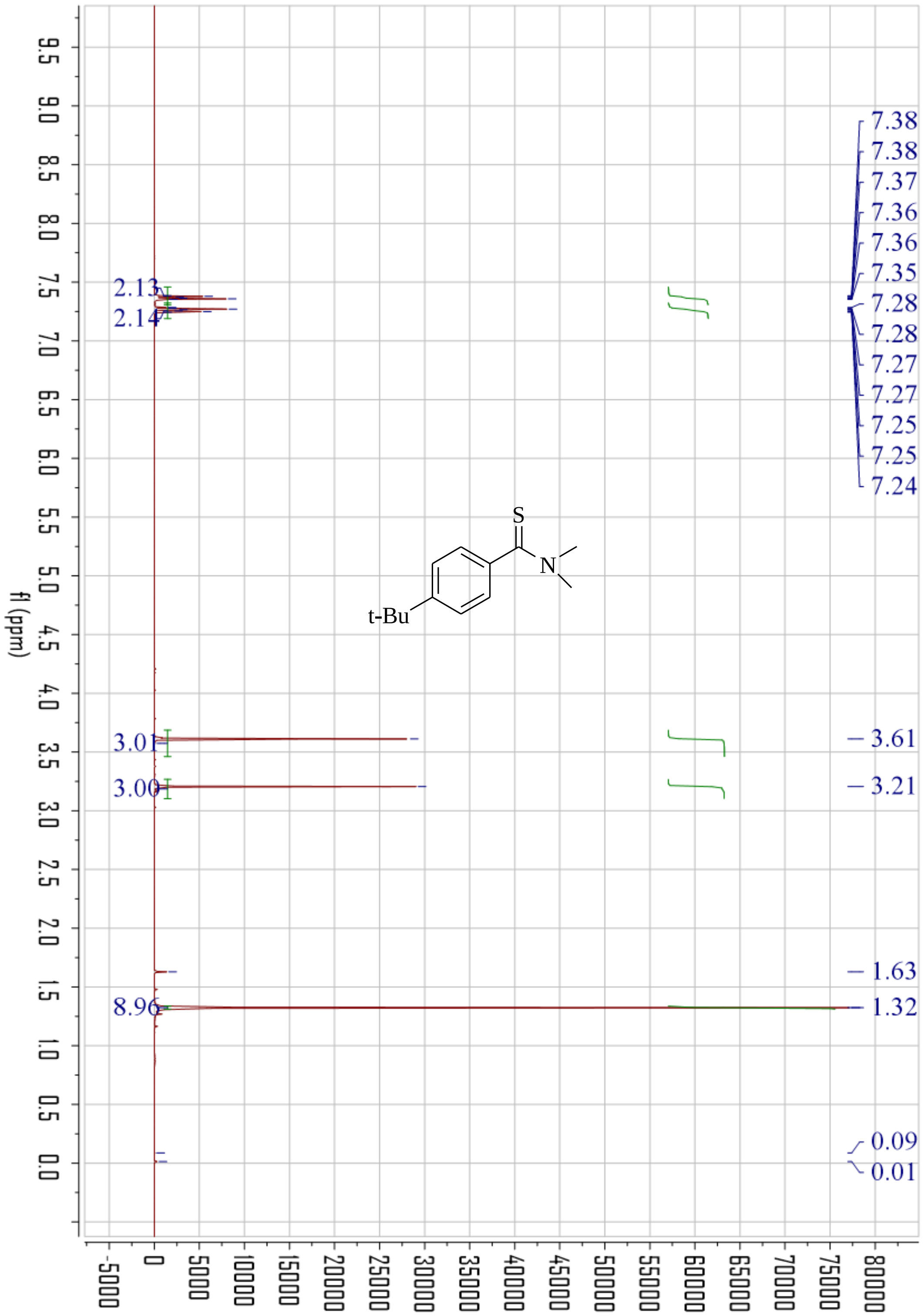

$^{13}$C NMR of 4-tert-butyl-*N*, *N*-dimethylbenzothioamide **3k**

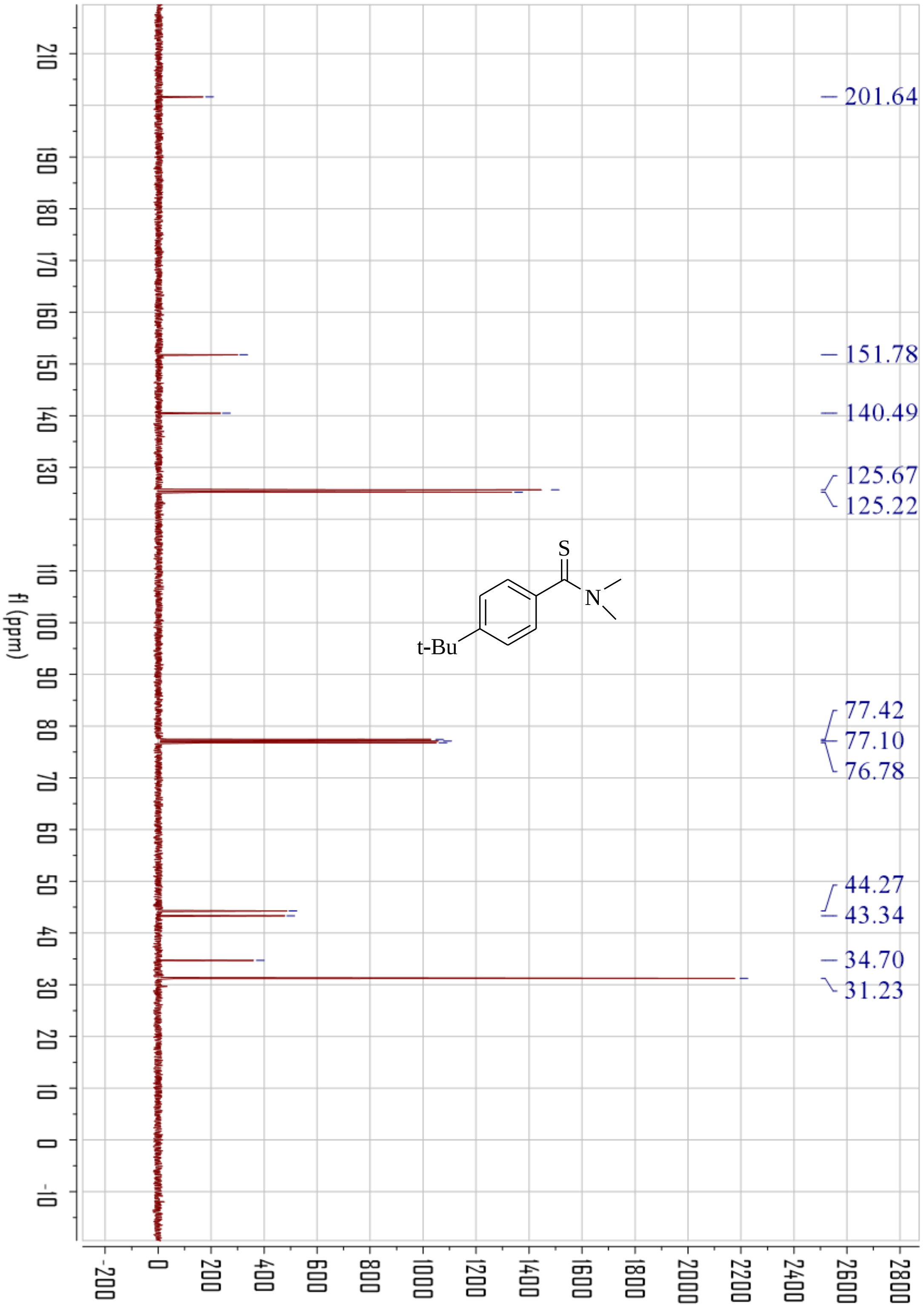

$^{1}$H NMR of 3,5-di-tert-butyl-*N*, *N*-dimethylbenzothioamide **3l**

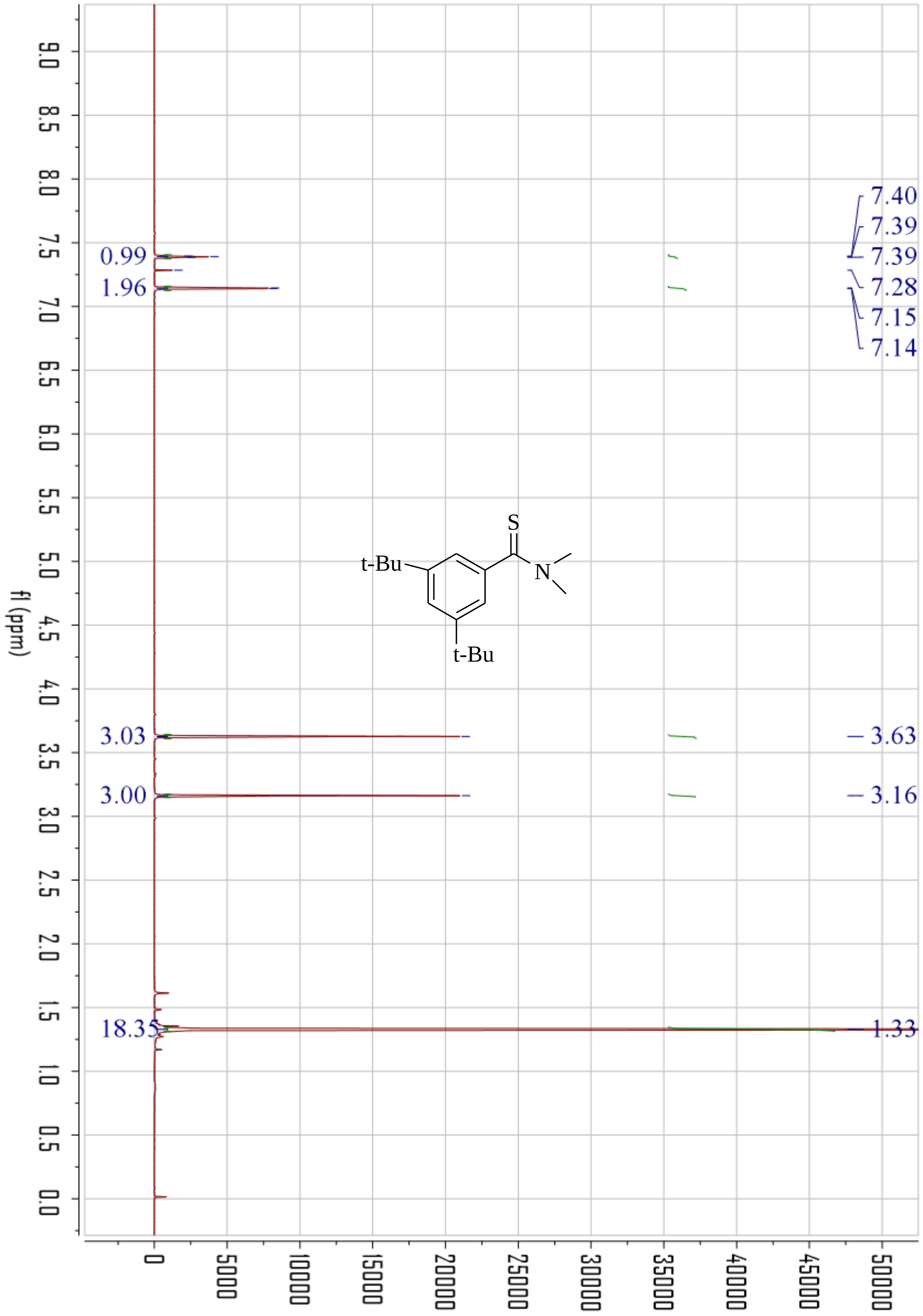

$^{13}$C NMR of 3,5-di-tert-butyl-*N*, *N*-dimethylbenzothioamide **3l**

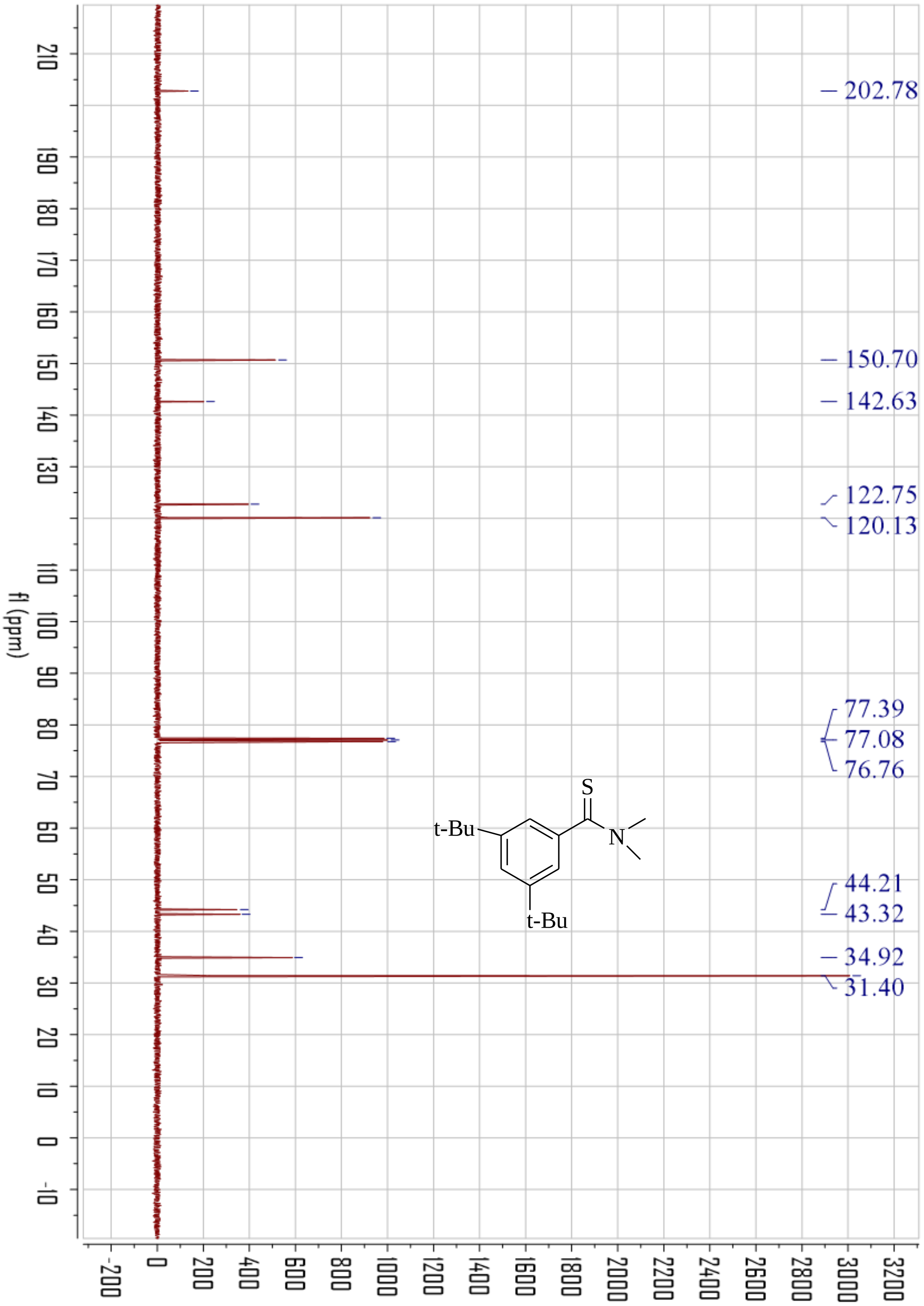

$^{1}$H NMR of 4-benzyl-*N*, *N*-dimethylbenzothioamide **3n**

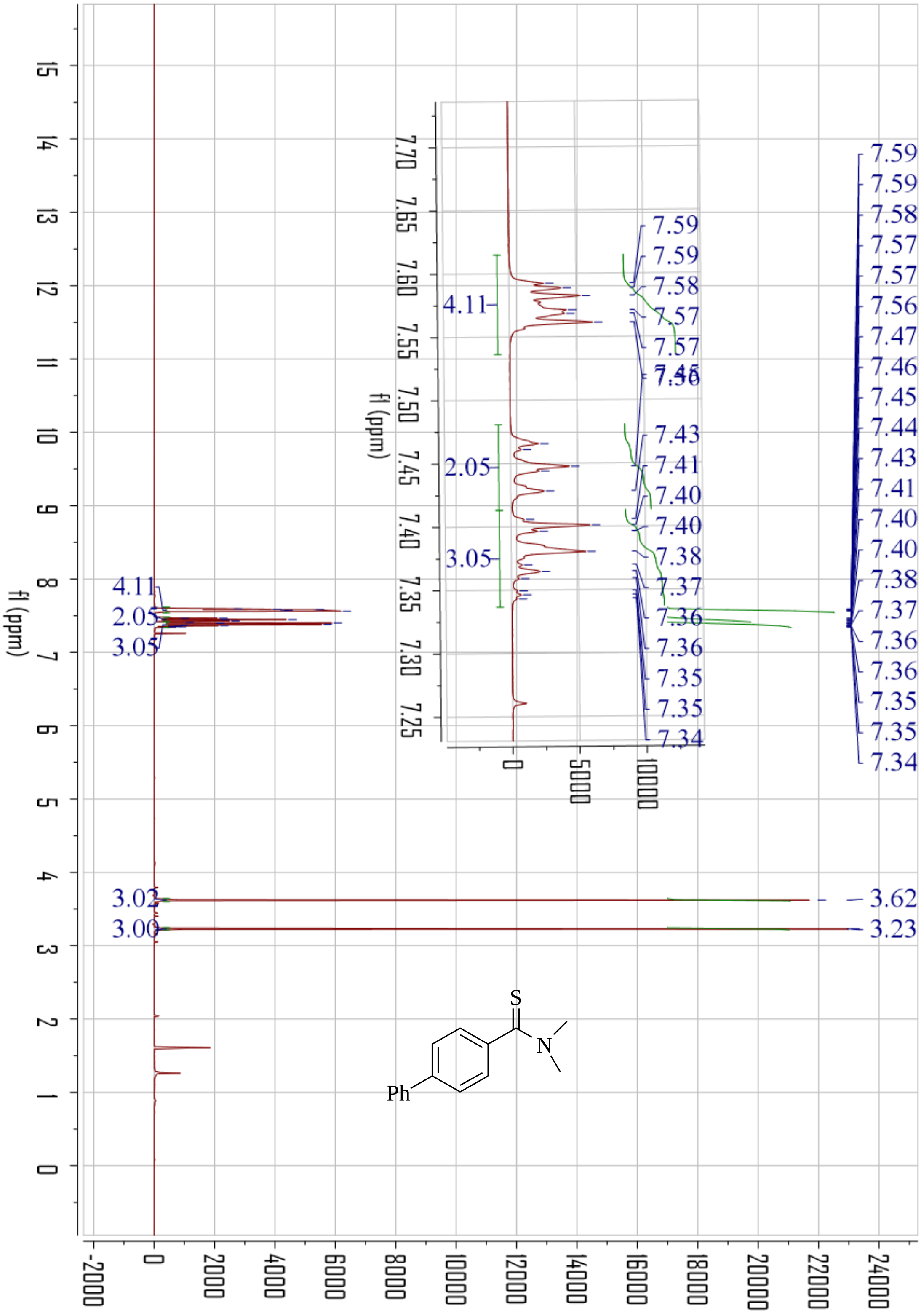

$^{13}$C NMR of 4-benzyl-*N*, *N*-dimethylbenzothioamide **3n**

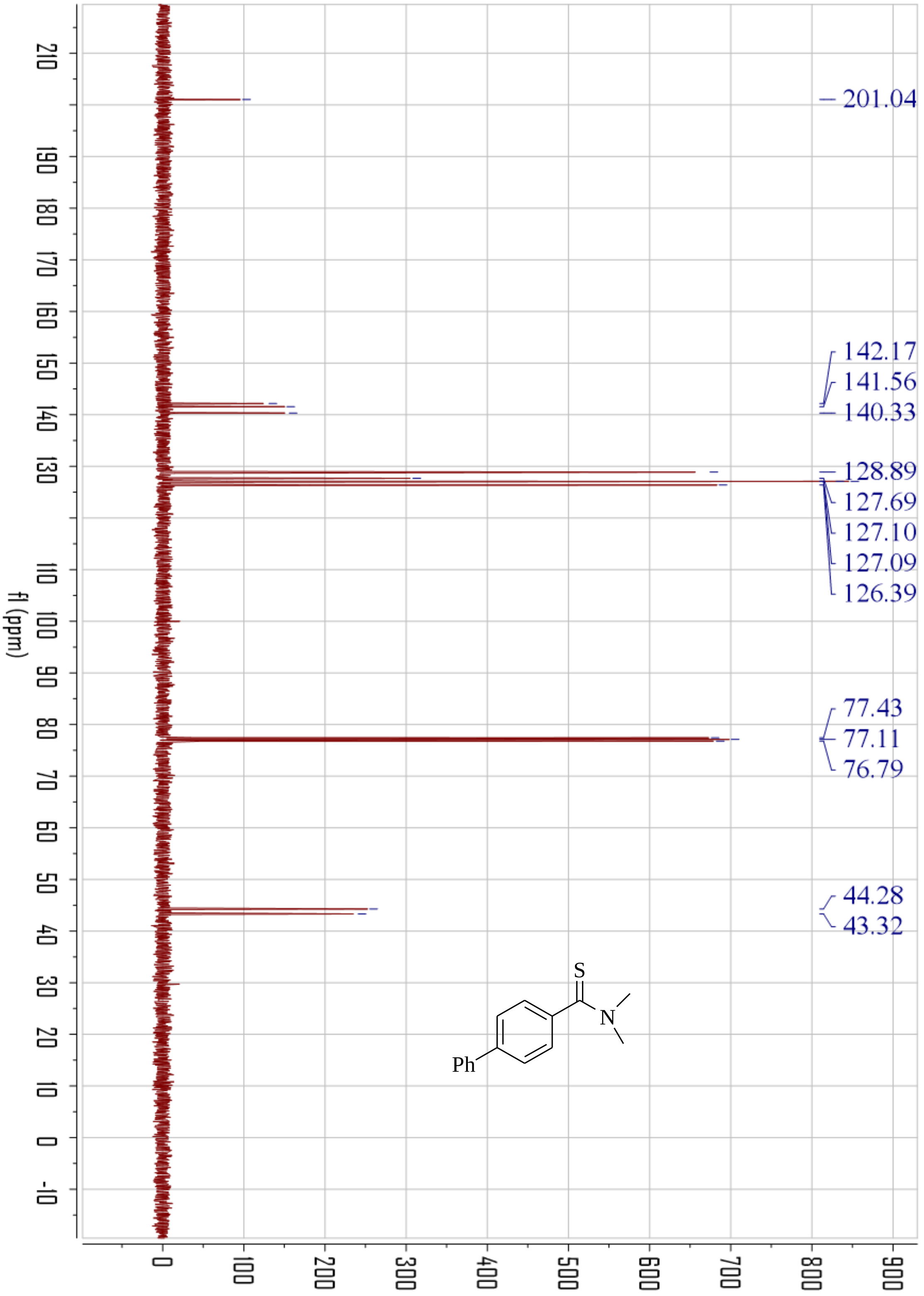

$^{1}$H NMR of 4-hydroxy-*N*, *N*-dimethylbenzothioamide **3o**

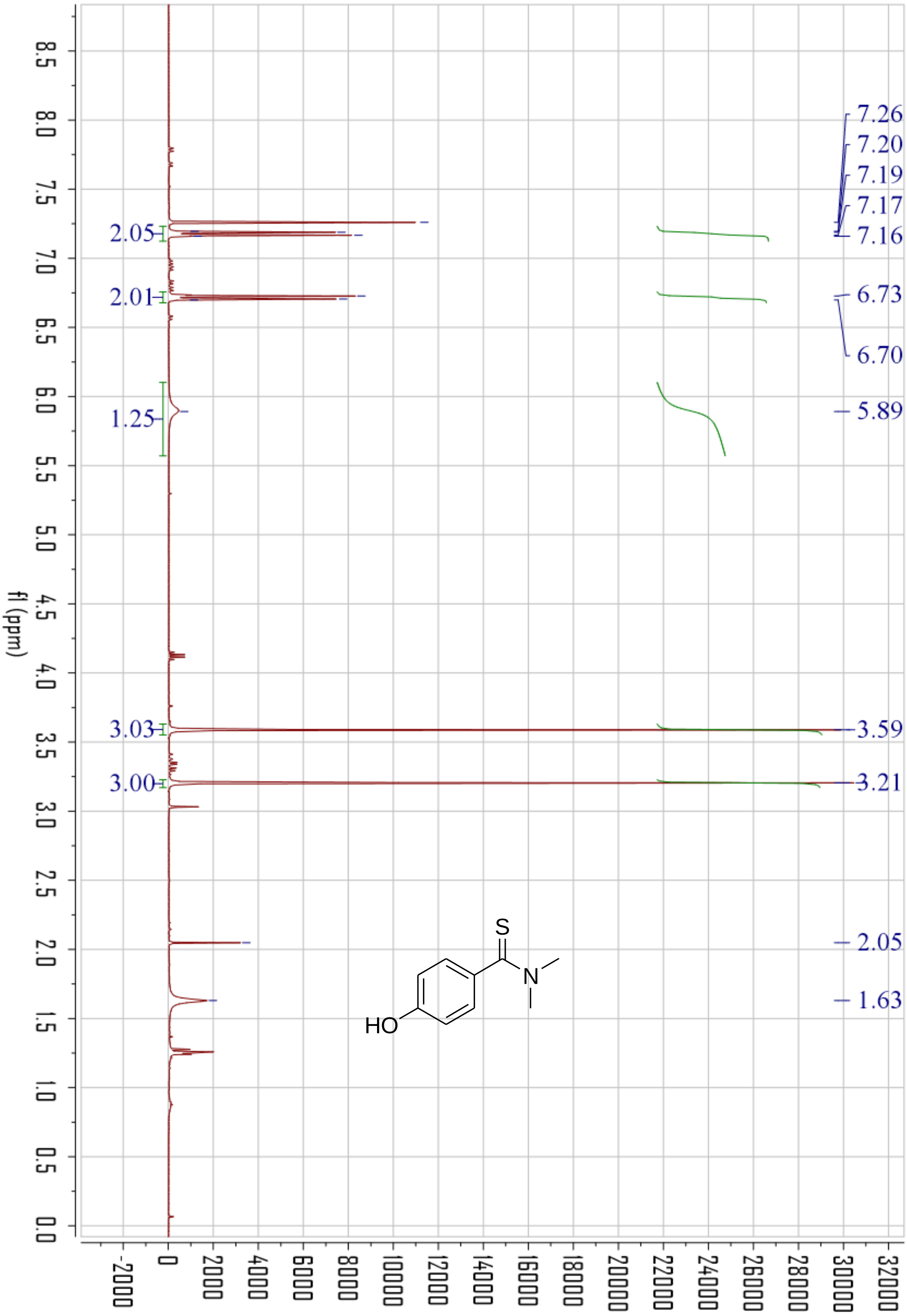

$^{1}$H NMR of 4-(dimethylamino)-*N*, *N*-dimethylbenzothioamide **3p**

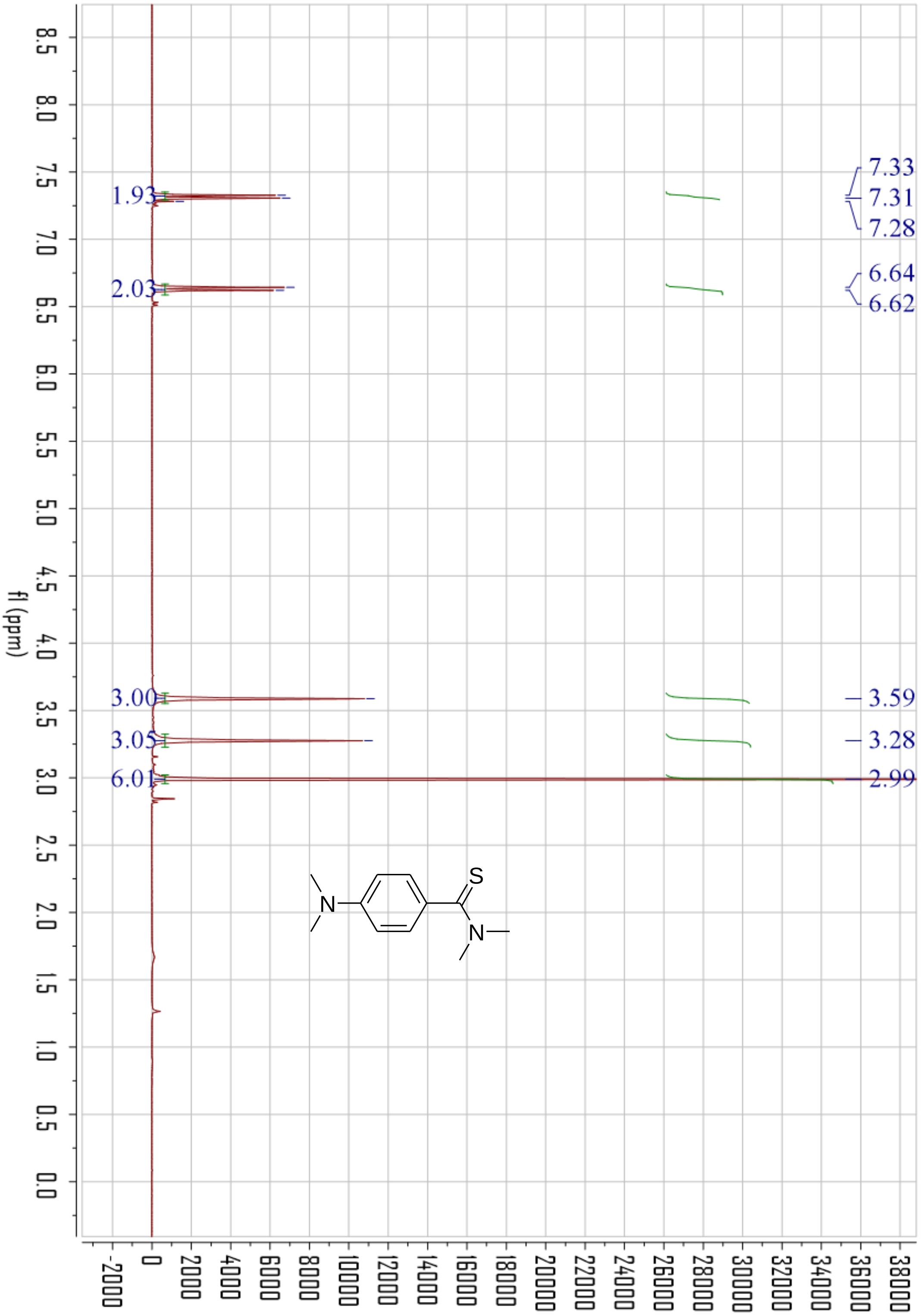

$^{13}$C NMR of 4-(dimethylamino)-*N*, *N*-dimethylbenzothioamide **3p**

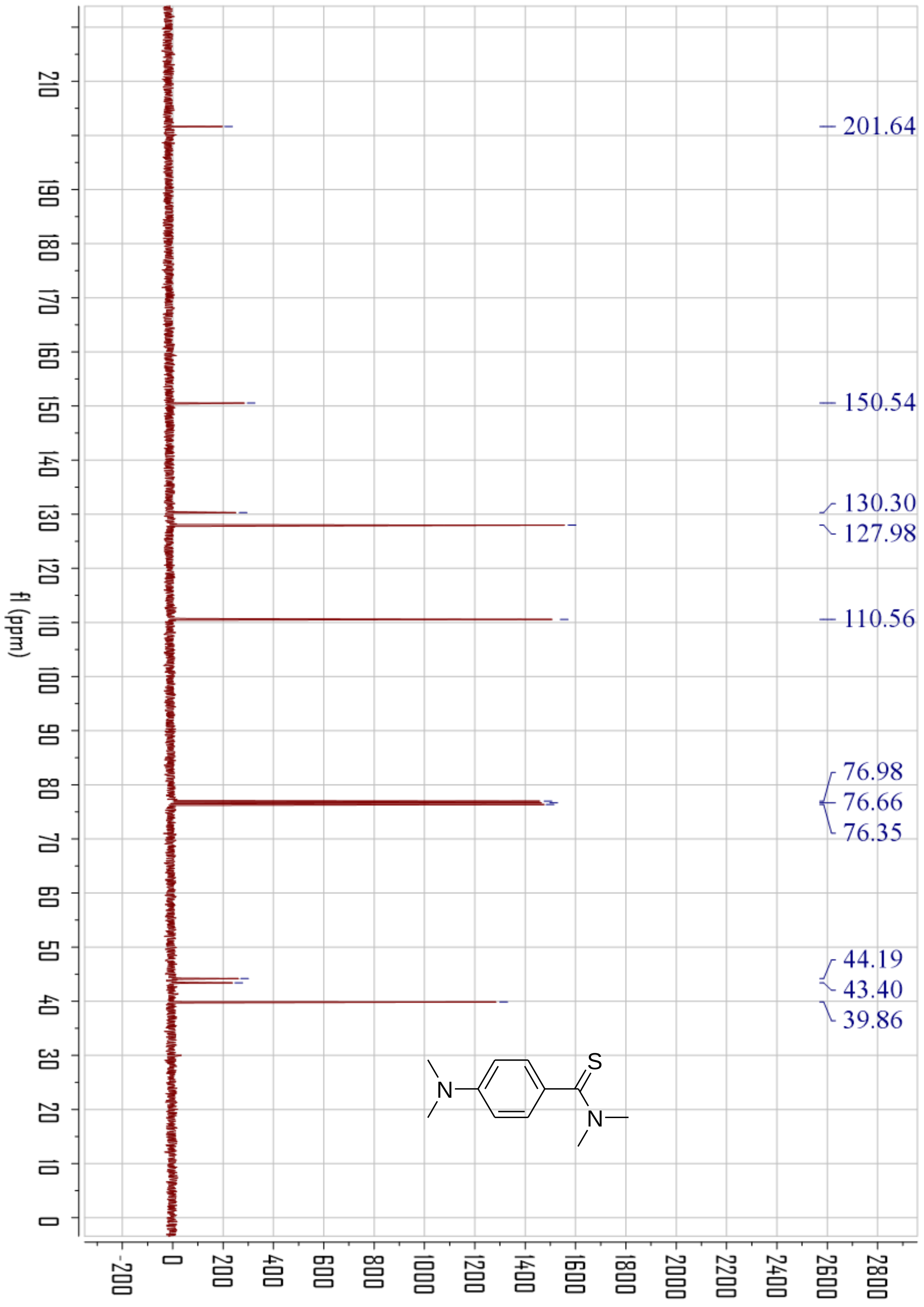

$^{1}$H NMR of *N*, *N*-dimethylnaphthalene-2-carbothioamide **3q**

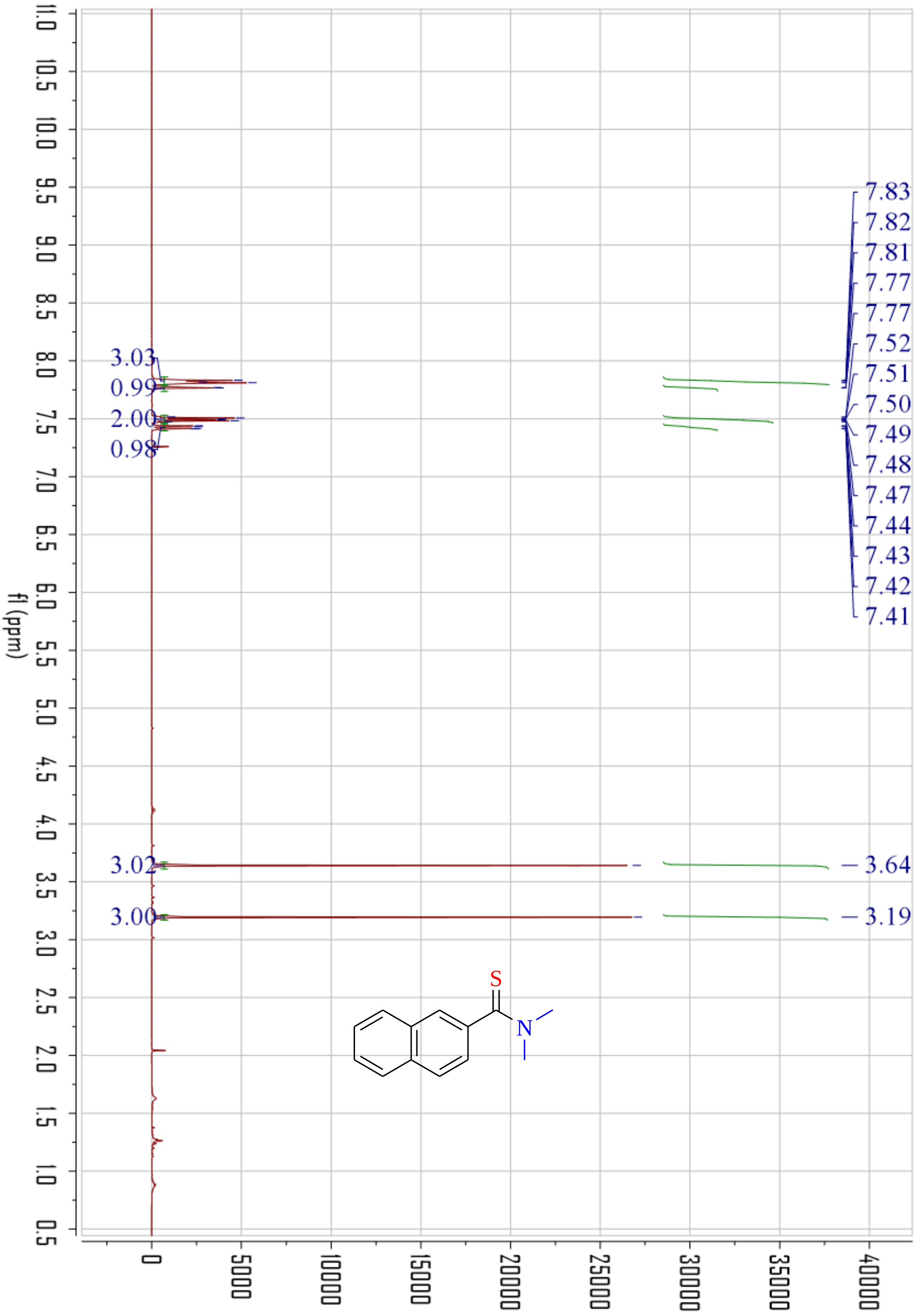

$^{13}$C NMR of *N*, *N*-dimethylnaphthalene-2-carbothioamide **3q**

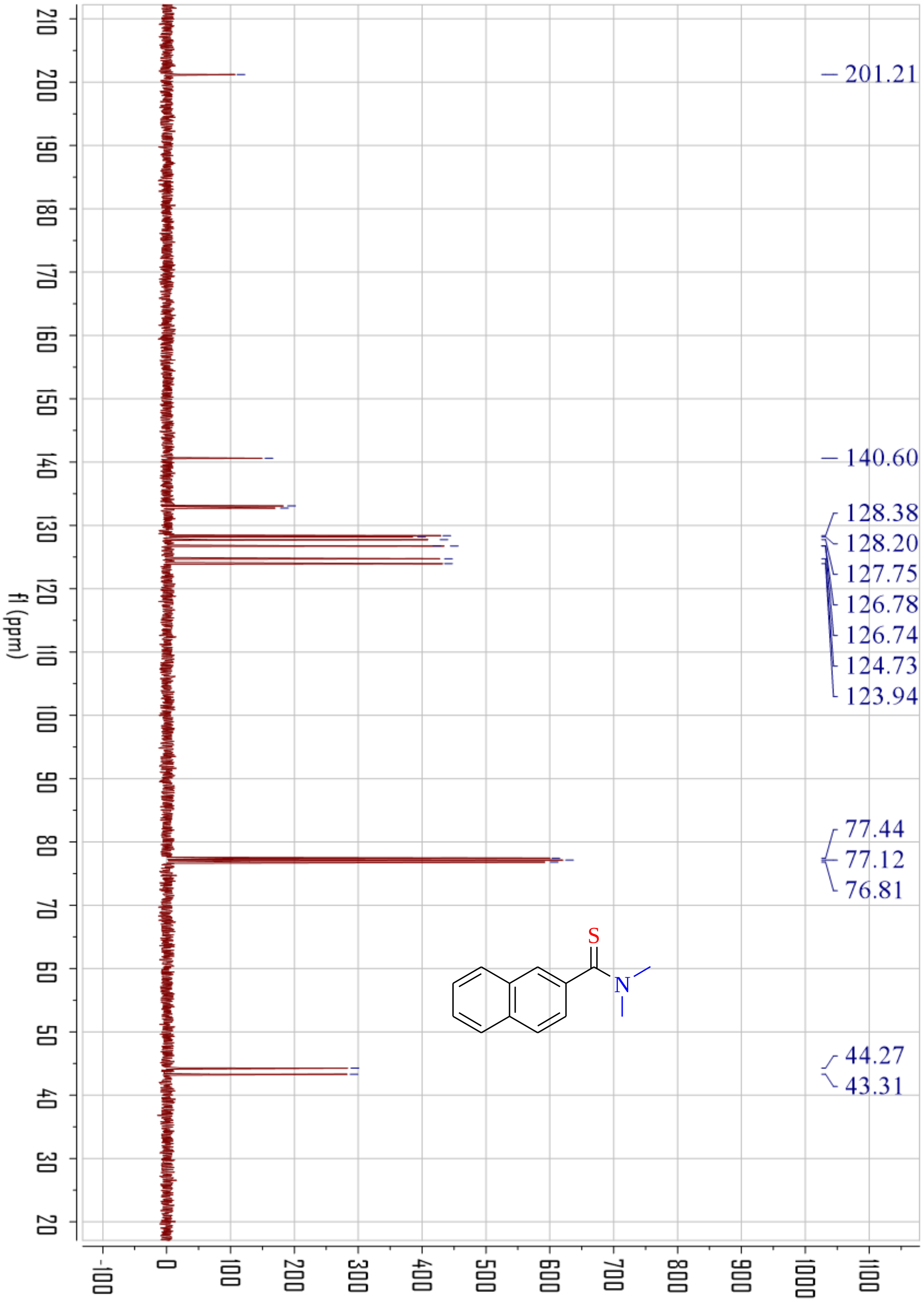

$^{1}$H NMR of *N*, *N*-dimethylfuran-2-carbothioamide **3s**

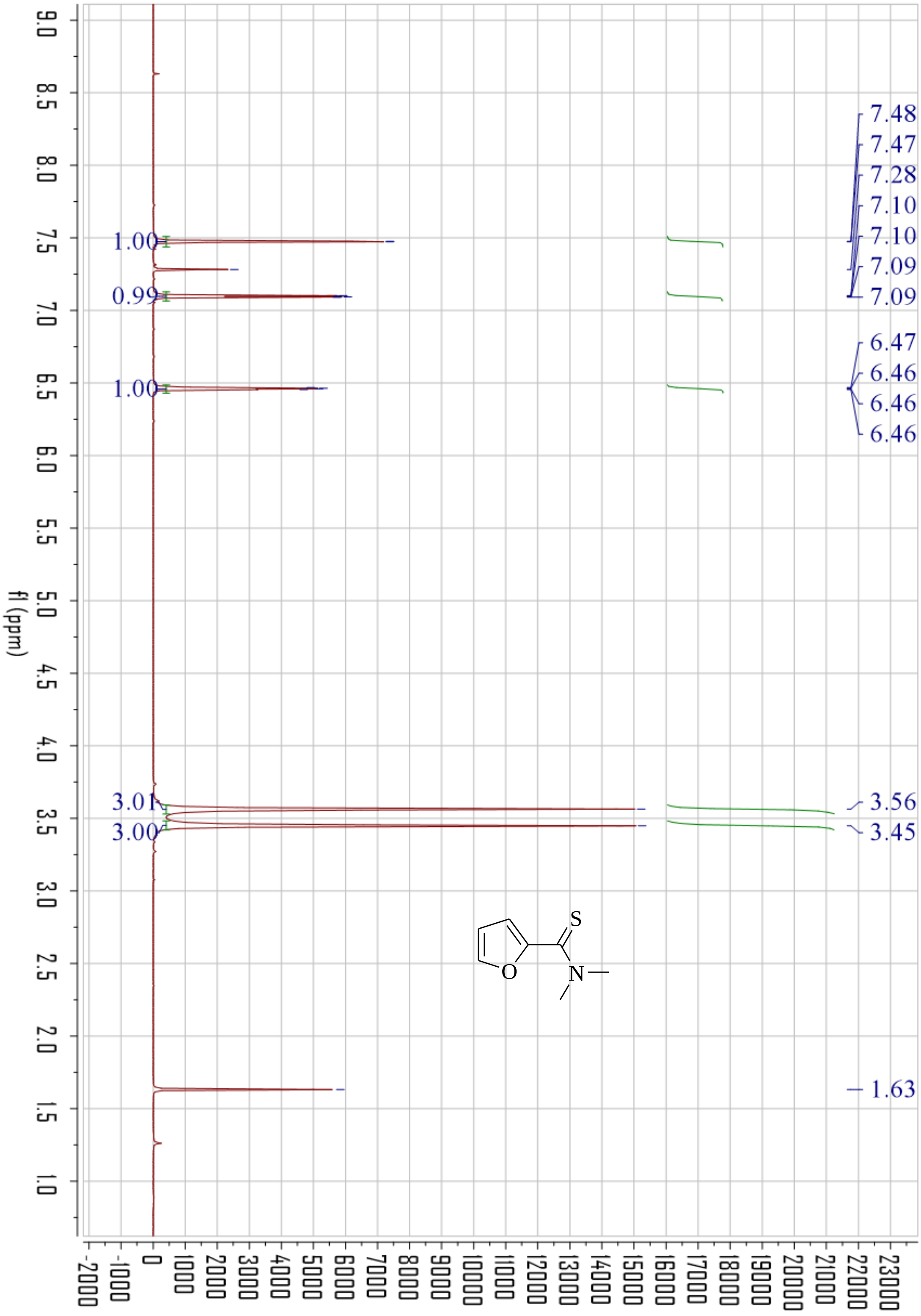

$^{13}$C NMR of *N*, *N*-dimethylfuran-2-carbothioamide **3s**

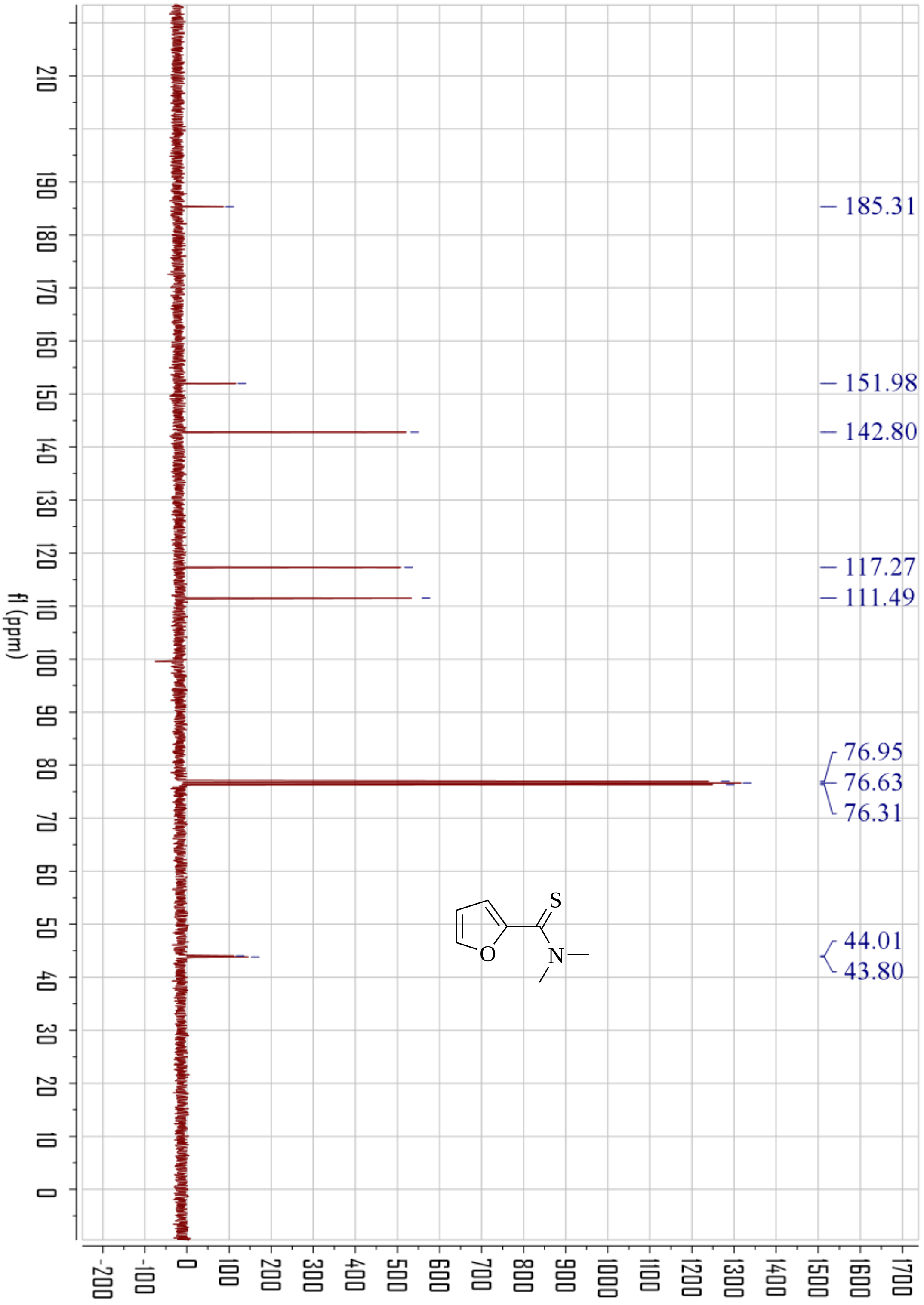

$^{1}$H NMR of *N*, *N*-dimethylthiophene-2-carbothioamide **3t**

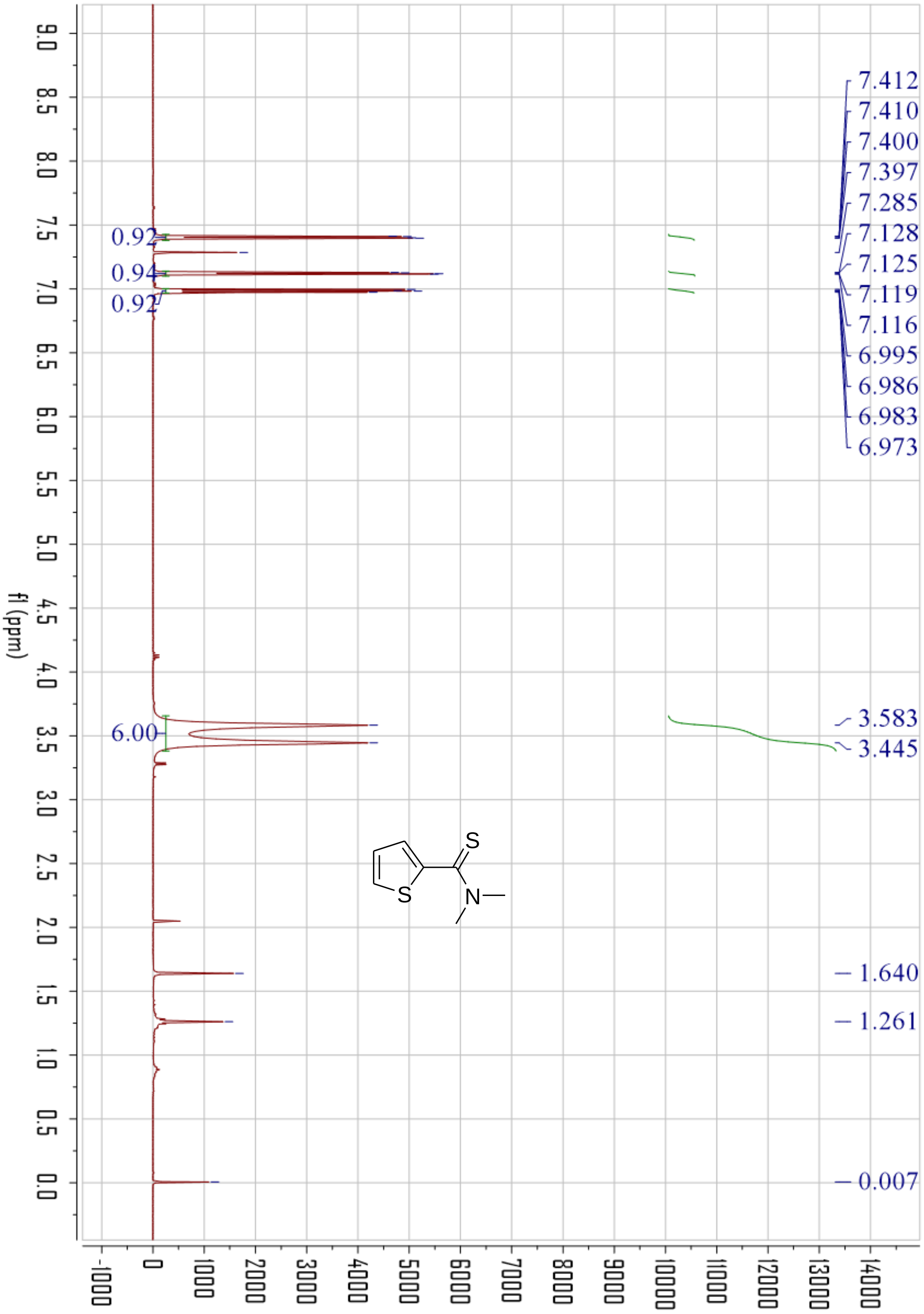

$^{13}$C NMR of *N*, *N*-dimethylthiophene-2-carbothioamide **3t**

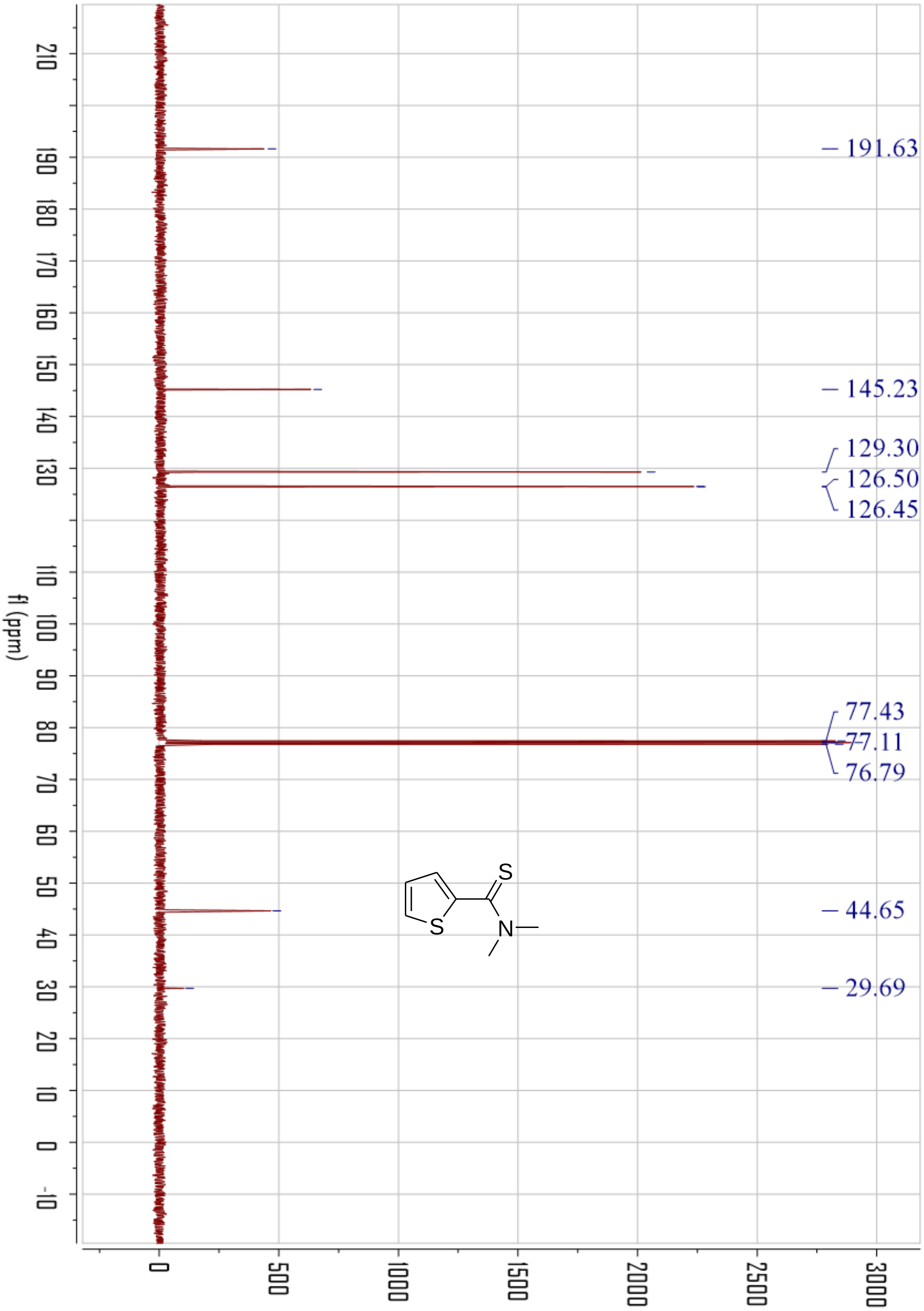

$^{1}$H NMR of *N*, *N*-dimethylpyridine-3-carbothioamide **3u**

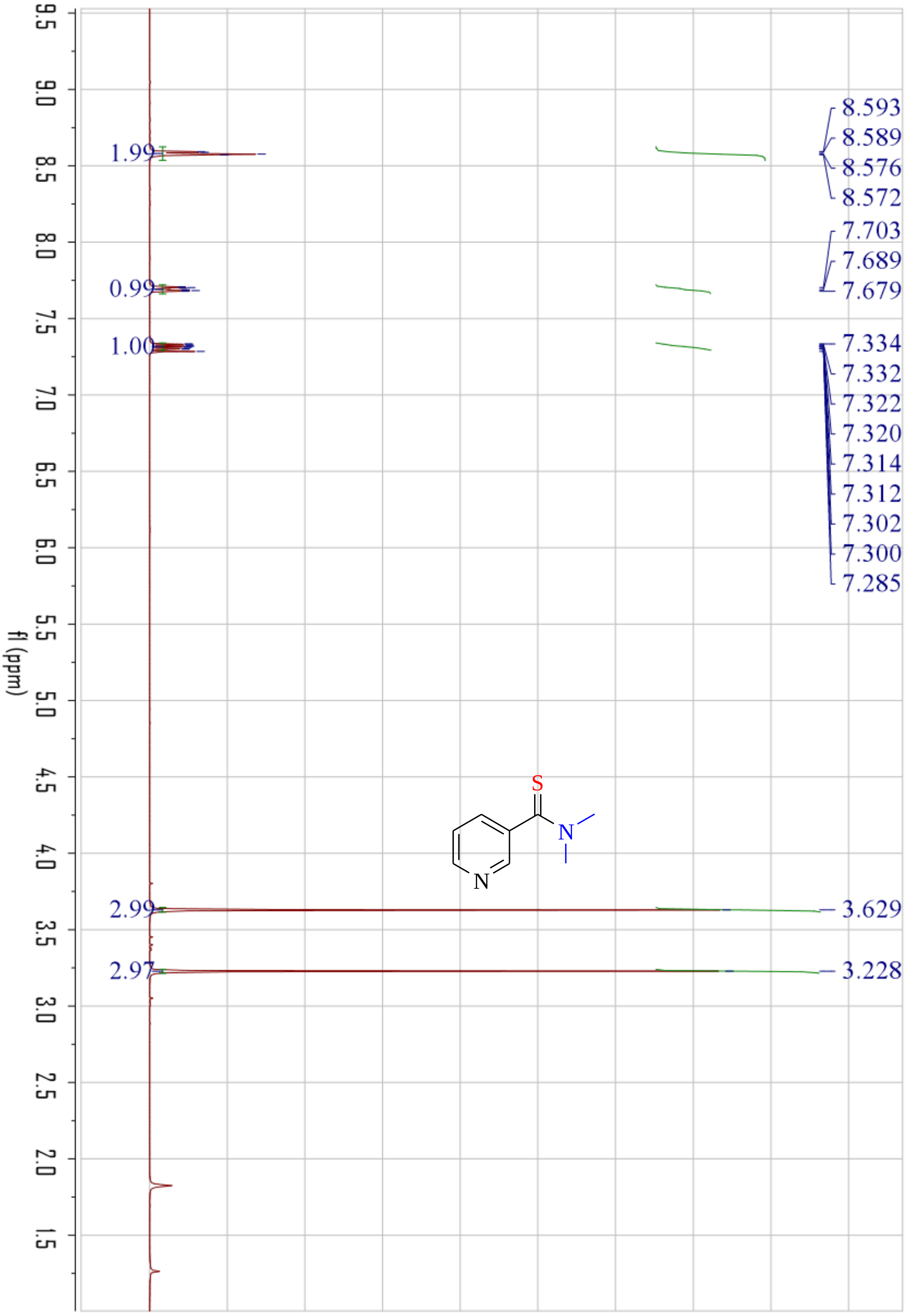

$^{13}$C NMR of *N*, *N*-dimethylpyridine-3-carbothioamide **3u**

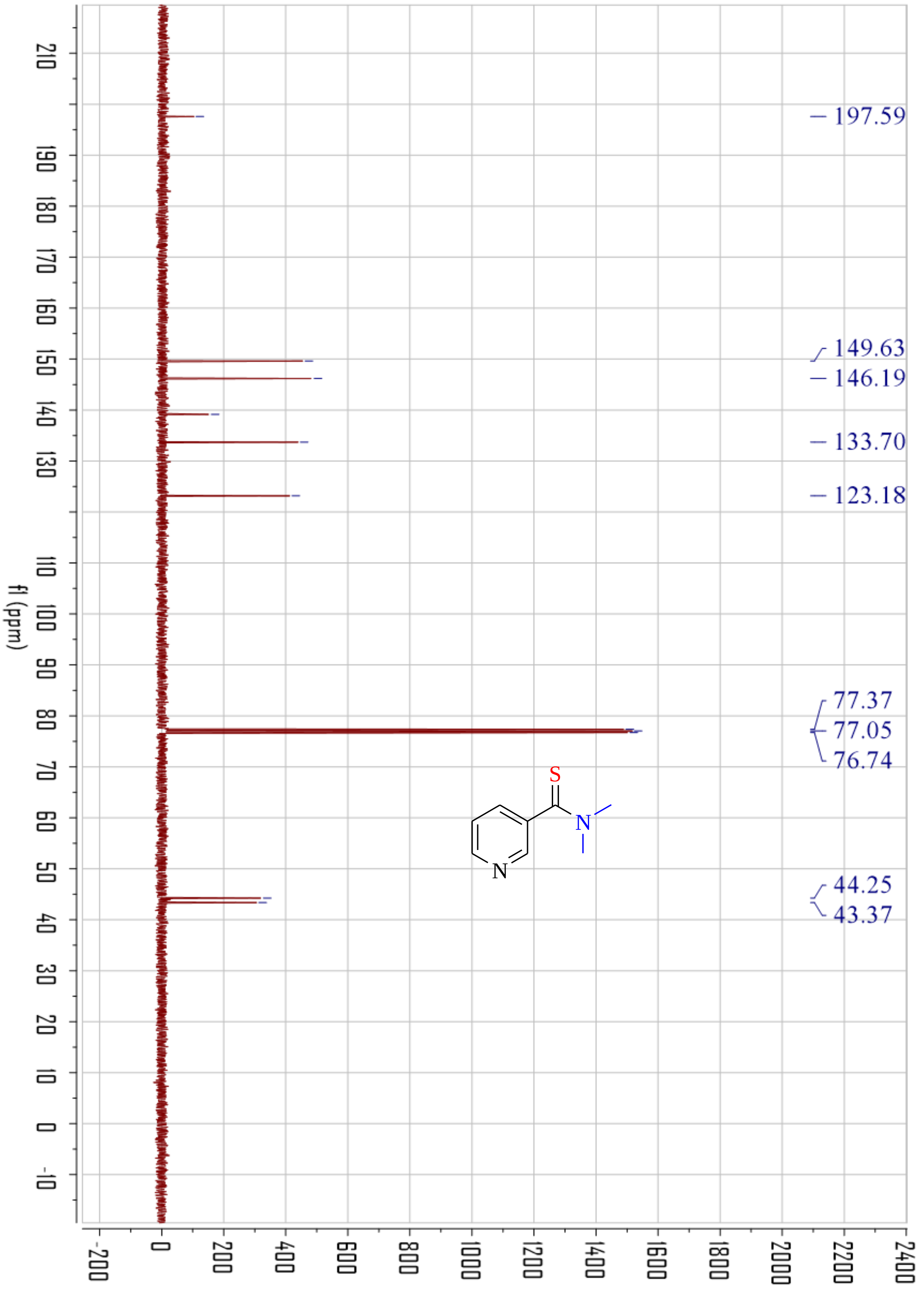

$^{1}$H NMR of *N*, *N*-dimethyl-5-(quinolin-2-yl)thiophene-2-carbothioamide **3v**

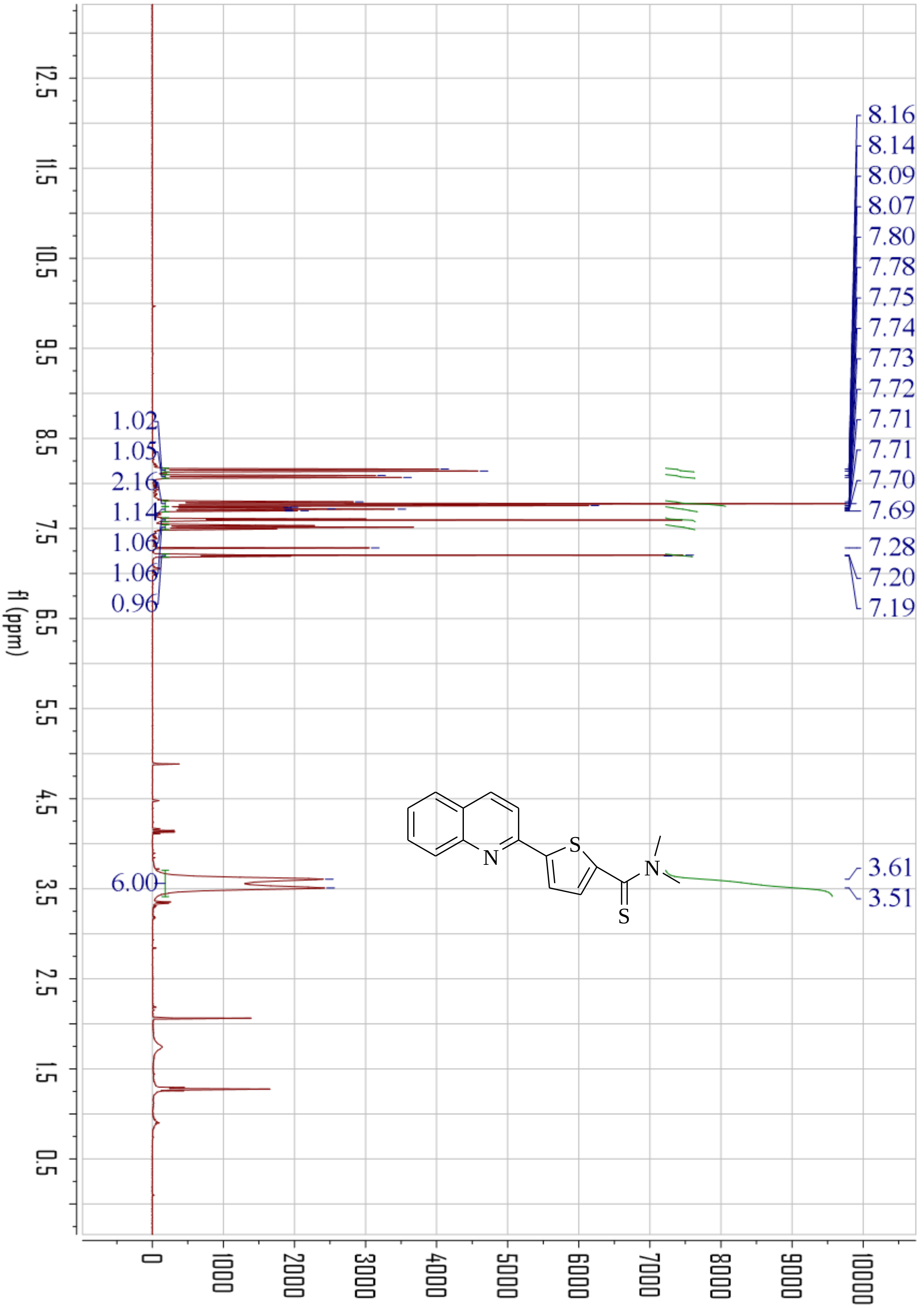

$^{13}$C NMR of *N*, *N*-dimethyl-5-(quinolin-2-yl)thiophene-2-carbothioamide **3v**

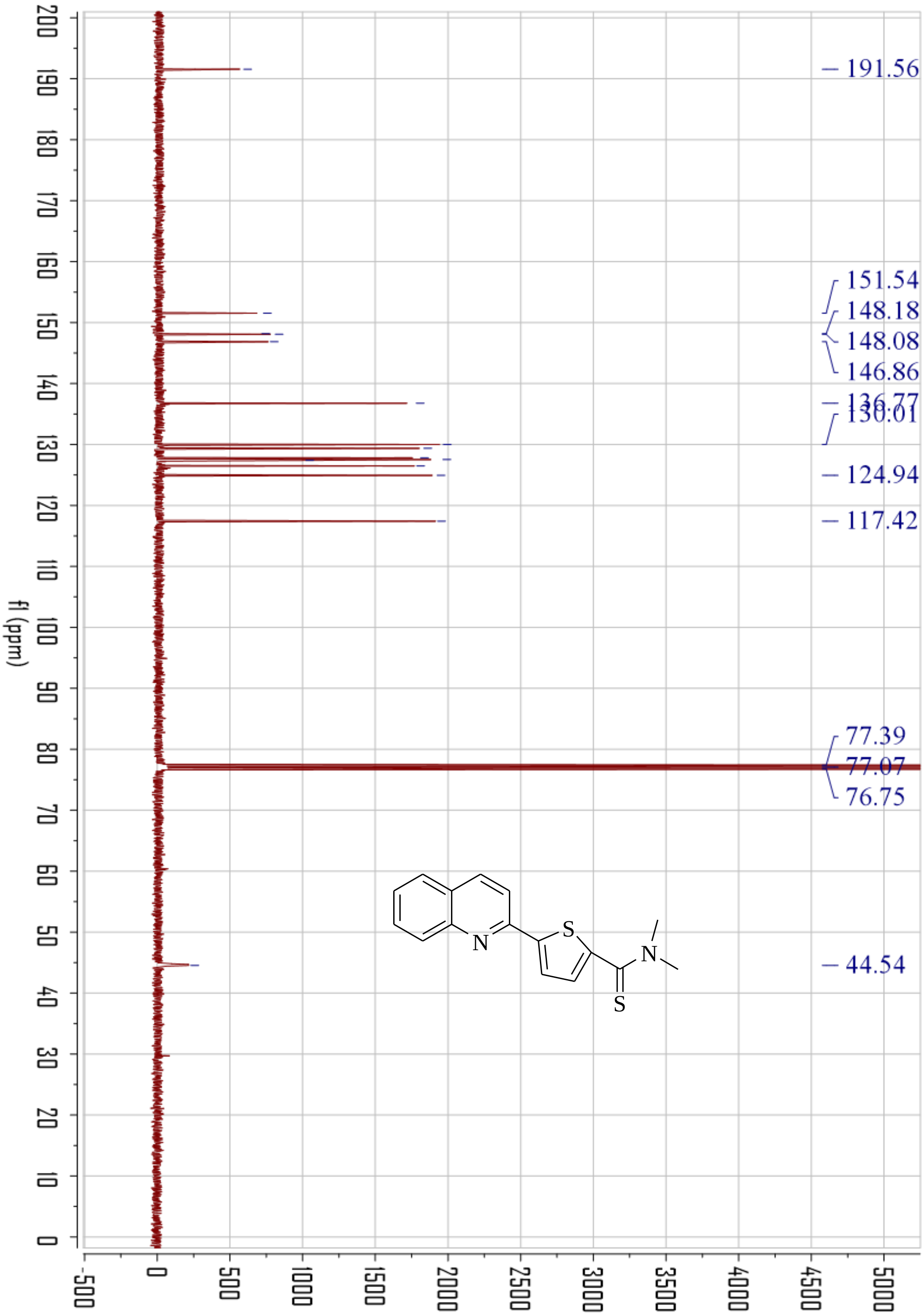

Supplement: Supplementary file 1 [file molecules-23-02225-s001.pdf]
